# An Updated Checklist of the Phytophagous Ladybird Beetles (Coccinellinae: Epilachnini) of China

**DOI:** 10.3390/insects17050450

**Published:** 2026-04-24

**Authors:** Muhammad Asghar Hassan, Bing-Lan Zhang, Zafar Iqbal, Muhammad Ali, Yi-Fei Sun, Taslima Sheikh, Hao-Sen Li, Hong Pang

**Affiliations:** 1 State Key Laboratory of Biocontrol, School of Ecology, Sun Yat-sen University, Shenzhen 518000, China; kakojan112@gmail.com (M.A.H.); sunyf28@mail2.sysu.edu.cn (Y.-F.S.); 2 School of Life Sciences/The Museum of Biology, Sun Yat-sen University, Guangzhou 510275, China; zhbingl@mail.sysu.edu.cn; 3 Key Laboratory of the Conservation and Exploitation of Biological Resources, College of Life Sciences, Anhui Normal University, Wuhu 241000, China; iqzafarshah@gmail.com; 4 Department of Zoology, University of Baltistan, Skardu 16100, Gilgit-Baltistan, Pakistan; muhammad.ali@uobs.edu.pk; 5 Department of Zoology, Government Degree College, Kathua 184101, India; sheikhtass@gmail.com

**Keywords:** biodiversity, Coccinelloidea, classification, biogeographical distribution, herbivory, host plant interaction, insect pest, ladybugs, taxonomic review, species list

## Abstract

A comprehensive annotated checklist of the members of the phytophagous ladybird beetle tribe Epilachnini (Coccinellinae) in China is compiled based on existing published sources and incorporates the latest taxonomic and nomenclatural updates. The checklist documents 176 extant species across 10 genera and provides analyses of regional species richness, distribution, and host plant associations. Regarding regional species richness, Yunnan Province is home to the highest number of species (76), followed by Taiwan (50), Sichuan (48), Guizhou (48), Guangxi (43), Tibet (43), Guangdong (25), Hainan (17), Hubei (17), Hunan (13), Shaanxi (13), Fujian (12), Henan (10), Jiangsu (10), Anhui (7), Shandong (7), Zhejiang (7), Jiangxi (5), Hong Kong (5), Gansu (5), Beijing (4), Hebei (4), Liaoning (3), Shanxi (2), and Chongqing, Jilin, Heilongjiang, Ningxia, and Xinjiang (each with one species). Among the recognized genera, *Epilachna* Chevrolat, 1837, is currently the most species-rich genera, with 59 species, followed by *Afissa* Dieke, 1947 (34), *Uniparodentata* Wang & Cao, 1993 (28), *Henosepilachna* Li, 1961 (29), *Afidentula* Kapur, 1958 (10), *Diekeana* Tomaszewska & Szawaryn, 2015 (9), and *Epiverta* Dieke, 1947 (4). Additionally, *Afidenta* Dieke, 1947, *Cynegetis* Chevrolat, 1837, and *Subcoccinella* Agassiz & Erichson, 1845 are each represented by a single species. Host plant data are currently available for only 72 species (approximately 41% of the species recorded in China), which are associated with 177 plant species across 34 families. The most frequently recorded host plant families are Solanaceae (43 species), Cucurbitaceae (32), Urticaceae (15), Fabaceae (14), Asteraceae (14), and Poaceae (10), whereas each of the remaining 28 families comprises fewer than 10 host species. For 104 species (59% of the Chinese members of the tribe), host plant associations remain unknown, highlighting a substantial gap in our understanding of their feeding habits.

## Simple Summary

The tribe Epilachnini currently comprising 1126 identified species in 28 genera is distributed worldwide. It is a highly diverse group of herbivorous ladybird beetles, predominantly found in the Afrotropical, Neotropical, and Oriental regions. This study provides a comprehensive checklist of 176 species across 10 genera, along with detailed data on their regional distribution and host plant associations in China. A significant ecological knowledge gap is revealed, as host plant associations are documented for only 72 species (41%) of the Chinese Epilachnini, which are primarily associated with the families Solanaceae, Cucurbitaceae, Urticaceae, Fabaceae, Asteraceae, Poaceae, Lamiaceae, Rubiaceae, Ranunculaceae, and Brassicaceae. The host plants of the remaining 59% of ladybird beetle species in this tribe remain unknown.

## 1. Introduction

The family Coccinellidae (Coleoptera), commonly known as ladybird beetles, ladybugs or lady beetles, is a charismatic and globally distributed group of insects, comprising approximately 6900 described species belonging to 360 genera in 25 tribes [1,2,3,4]. Currently, the family is divided into three subfamilies [3]: Coccinellinae (336 genera, more than 6500 species), Microweiseinae (23 genera, more than 150 species) and Monocoryninae (1 genus, 10 species) [4,5,6,7]. Ladybird species are predominantly recognized for their predatory nature, with over half of the family preying on various insect groups, particularly scale insects (36%) and aphids (20%), thereby serving as vital biological control agents [8]. However, beyond this familiar predatory stereotype, Coccinellidae exhibits a significant diversity of feeding habits. Approximately 20% of ladybird species are phytophagous. This group includes members of the tribe Epilachnini, which consists of around 1126 formally described extant species classified into 27 recognized genera. Furthermore, the genus *Bulaea* Mulsant, 1850 (comprising four species), which belongs to the tribe Coccinellini, is also phytophagous [1,2,9,10,11]. A smaller proportion, approximately 2%, are mycophagous, while others feed on pollen and small arthropods [1,12].

Epilachnini represents the largest phytophagous group of herbivorous beetles within the family Coccinellidae. Notably, several species within this tribe are of major agricultural importance. For example, *Epilachna varivestis* Mulsant, 1837 (commonly referred to as the Mexican bean beetle), is widespread in the Nearctic and Neotropical regions [1,13,14], while *Henosepilachna vigintioctopunctata* (Fabricius, 1775) (known as the 28-spotted ladybird or the Hadda beetle) is prevalent in the Oriental, Palaearctic, and Australian regions [1,15,16,17]. Additional economically significant pests include *Henosepilachna vigintioctomaculata* (Motschulsky, 1857) (also called the 28-spotted potato ladybird beetle), *H. implicata* (Mulsant, 1850), and *Chnootriba elaterii* (Rossi, 1794) (known as 12-spotted melon beetle) [1,16,18,19,20]. These species are known to infest a wide range of cultivated crops, including cucumber, ivy gourd, tomato, eggplant, melon, potato, pumpkin, snake gourd, tobacco, and ramie [18,20,21,22]. Despite the agricultural importance of several well-documented pest species, host plant associations remain poorly documented for the tribe. Currently, host plant associations are documented for only 134 species (approximately 12% of Epilachnini) [16,20,21,22,23,24,25,26,27], representing 15 genera (55.5% of the tribe). Consequently, host-plant associations remain unknown for 986 species, representing approximately 88% of the tribe. This substantial gap in knowledge highlights a significant deficiency in our understanding of feeding habits and host plant interactions within Epilachnini. Such information is fundamental to understanding the evolutionary history of phytophagous lineages, including patterns of host shifts and host-associated diversification, and is also important for predicting pest host ranges and developing effective management strategies [22].

In earlier taxonomic classifications, Epilachnini was treated as a distinct subfamily, Epilachninae, and was further divided into four tribes: Epilachnini Mulsant, 1846; Cynegetini Thomson, 1866; Epivertini Pang & Mao, 1979; and Eremochilini Gordon & Vandenberg, 1987 [1,13,28,29,30]. The designation Epilachnini was originally proposed by Costa in 1849 to include three species: *Epilachna chrysomelina* (Fabricius, 1775) (now referred to as *Chnootriba elaterii* (Rossi, 1794), *Cynegetis globosa* (=*Subcoccinella vigintiquatuorpunctata*), and *Epilachna argus* (Fourcroy, 1785) (now known as *Henosepilachna argus* (Geoffroy *in* Fourcroy, 1785)) [13]. Despite the earlier classification, recent taxonomic studies, which have examined larval and adult morphological characteristics, food preferences, and phylogenetic relationships, have consistently identified Epilachnini as a monophyletic clade within Coccinellinae [2,12,31,32,33,34].

The tribe Epilachnini currently comprises 27 recognized genera, of which two, *Afidenta* and *Macrolasia*, are monotypic, and the number of species in 23 genera ranges between two and seventy. Among these, *Epilachna* (with around 484 species) and *Henosepilachna* (233 species) are considered the most species-rich genera. However, the true diversity and taxonomic limits of *Epilachna* remain controversial. Once considered a cosmopolitan genus, *Epilachna* is now believed to be restricted to the New World, with many Asian species transferred or reassigned to other genera such as *Afissa*, *Diekeana*, and *Uniparodentata* [2,20,35,36]. *Epilachna* was the first described genus within phytophagous ladybird beetles and historically served as a “catch-all” genus for many newly described species. However, subsequent taxonomic revisions and a better understanding of morphological and phylogenetic relationships have led to establishment of several new genera within the tribe, and many species formerly assigned to *Epilachna* have been transferred to other genera. Despite these advances, a significant number of species originally described under *Epilachna* remain taxonomically unresolved. Although there has been considerable progress in clarifying generic boundaries and phylogenetic relationships within Epilachnini, many species originally described in *Epilachna* still require further taxonomic revision.

The Chinese fauna of Epilachnini currently comprises 10 genera and approximately 176 species and represents about 15% of the known global diversity of the tribe. The earliest record of Epilachnini from the territory of present-day China dates back to Mulsant [37], who described *Epilachna maculivestis* from Tibet. Thereafter, numerous species have been described from China over the past 170 years by many authors, including Crotch [38], Weise [39], Gorham [40], Frivaldszky [41], Sicard [42], Weise [43,44], Mader [45,46], Zimmermann [47], Liu [48], Li & Cook [21], Kapur [49], Bielawski [50], Miyatake [51], Pang & Mao [52,53], Hoàng [54], Cao & Xiao [55], Cao & Wang [56], Xiao & Li [57], Pang [58], Zeng & Yang [59,60], Hu & Zhang [61], Yu & Wang [62], Yu [63], Zeng [64,65], Peng et al. [66], Pang & Zeng [67], Yu [68], Pang et al. [69], Yi et al. [70], Wang et al. [71], Tomaszewska et al. [72], L. Wang et al. [73], Jin et al. [74], and Ye et al. [75]. A list of 106 *Epilachna* species recorded in China was relatively recently compiled by Pang et al. [69]. Since then, however, many taxonomic changes have been made and many new species described.

Since the publication of the world catalog of Epilachnini by Jadwiszczak & Węgrzynowicz [1], the taxonomy of this tribe has undergone a significant revision, with several new genera established and numerous nomenclatural changes proposed. The present study aims to provide an updated checklist of the Chinese Epilachnini, summarizing current taxonomic knowledge and providing a foundation for future systematic and biodiversity studies of the tribe in the region.

## 2. Materials and Methods

The checklist present below includes the valid generic and species names of Epilachnini found in China, their synonyms, as well as their regional and global distributions, and associated host plant data published up to 1 April 2026. All the data presented here has also been entered into the online database LadybirdBase (http://www.ladybirdbase.com/home/, accessed on 1 January 2026), ensuring wide accessibility and integration with global ladybird taxonomic resources.

### 2.1. Taxonomic Data Sources

Valid species names and their synonyms were derived from the following publications: Korschefsky [76], Fürsch [77,78,79,80], Kapur [49,81,82,83,84,85,86], Li & Cook [21], Gordon [13], Hoàng [54,87,88], Pang & Mao [53], Miyatake [51,89], Gordon & Vandenberg [30], Wang & Cao [90,91,92], Katakura et al. [23], Jadwiszczak & Węgrzynowicz [1], Poorani [93], Kovář [94], Ślipiński [31], Ren et al. [9], Szawaryn [95,96], Canepari [15], Pang et al. [69], Tomaszewska & Szawaryn [11], Wang et al. [71,73], Szawaryn et al. [34], Tomaszewska & Szawaryn [2], Tomaszewska et al. [72], González [97], González et al. [98], Bajracharya & Budha [99], Iqbal et al. [20], Duarte-de-Mélo et al. [17], Wang et al. [73], Jin et al. [74], and Ye et al. [75].

### 2.2. Classification and Taxonomic Arrangements

The taxonomic classification adopted herein is based on the recent classification systems proposed by Szawaryn et al. [34] and Tomaszewska & Szawaryn [2]. The classification has been updated to incorporate all subsequent taxonomic changes published between 2016 and 2025, including re-validation of previously synonymized taxa, descriptions of new taxa, and newly proposed synonyms and combinations (see references above). These updates were further verified using online biodiversity platforms and specialized databases, including the Global Biodiversity Information Facility (GBIF: https://doi.org/10.15468/39omei, accessed on 1 January 2026) and Catalog of Life (https://doi.org/10.48580/dgwnl, accessed on 1 March 2026), LadybirdBase [4], and Coccinellidae South America [100]. All known genera and species are listed alphabetically in the checklist.

### 2.3. Geographical Distribution

The geographical distribution data are presented as follows: biogeographical region, followed by China (with provinces in parentheses), and other countries listed alphabetically within each region. Here we follow the traditional zoogeographical classification of China into the Palaearctic and Oriental regions. The Palaearctic region includes northern and western China, whereas the Oriental region comprises southern and eastern China. The boundary between these two regions in eastern China is generally considered to follow the Qinling Mountains–Huaihe River line. In western China, however, this boundary is less distinct and forms a broad transitional belt encompassing southern Gansu, northwestern Sichuan, northwestern Yunnan, and southeastern Tibet. Therefore, provinces located within this transitional zone, such as parts of Sichuan, Yunnan, and Tibet, should be assigned with caution, as species in these regions may show affinities with both regions. The transition zone of these two regions have been discussed for various groups of animals [101,102,103,104]. Herein we currently assign Sichuan and Yunnan in the Oriental region, and Tibet in the Palaearctic region.

**Figure 1:**
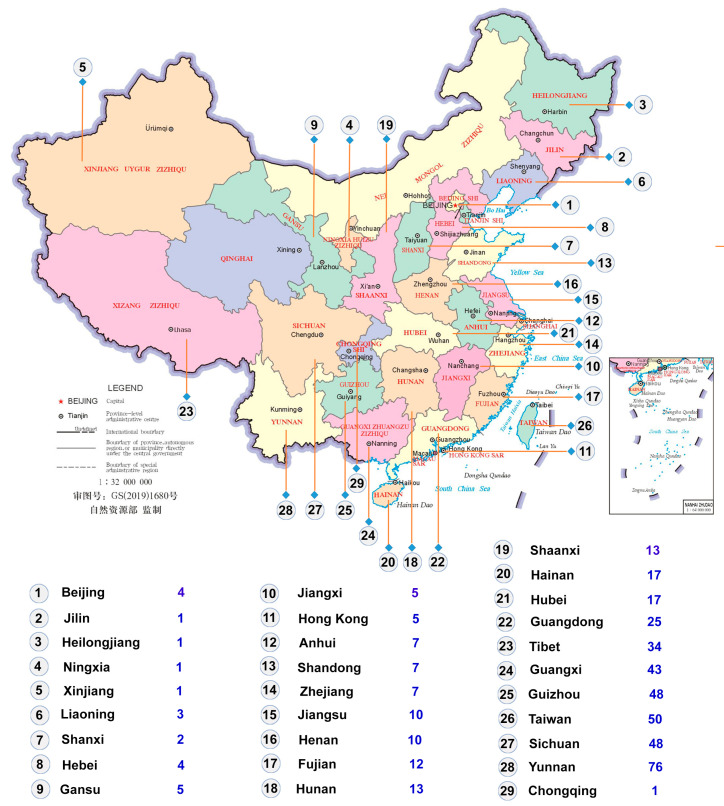
Regional distribution of the phytophagous ladybird beetle species in China. The numbers in gray circles represent regions and the blue numbers indicate the number of recorded species in each region. Source (http://bzdt.ch.mnr.gov.cn/browse.html) accessed on 15 April 2026.

The map of China shown in Figure 1 was obtained from the Ministry of Natural Resources Standard Map Service website GS (2019) 1680, accessed on 20 January 2026 (http://bzdt.ch.mnr.gov.cn/). Detailed host plant records for individual species are provided in the annotated checklist.

### 2.4. Host Plant Data

**Figure 2:**
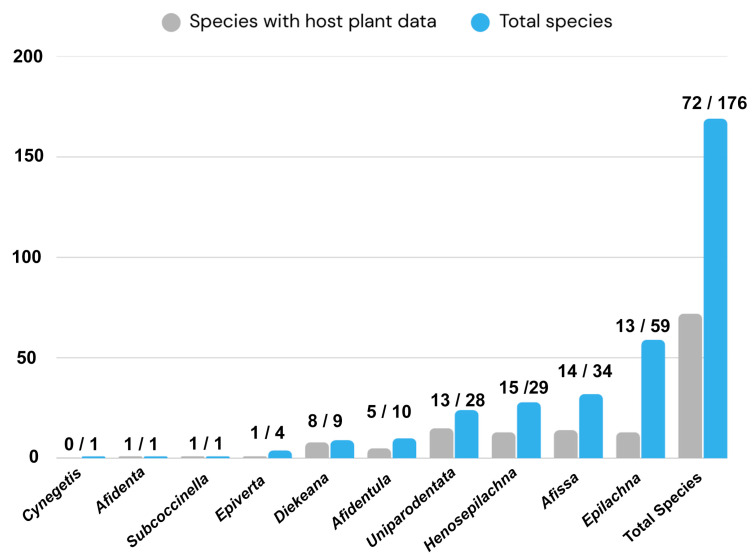
The number of species with a known host plant association compared with the total number of species within each genus of the Chinese Epilachnini.

Host plant associations are currently available for only 72 species, representing nine of the 10 genera in China (Figure 2). These species are associated with 177 plant species belonging to 34 families. Host plant data were compiled from an extensive review of historical and contemporary literature, including Pang & Mao [53], Cao [105], Singh & Phaloura [106], Wang & Cao [90], Zeng & Yang [59,60], Shirai & Katakura [107], Katakura et al. [23], Yu & Lau [108], Zhang & Ou [109], Naz et al. [110], Yi et al. [70], Katoh et al. [22], Casari & Teixeira [111], Ahmed et al. [112], Ohta-Matsubayashi et al. [24], Sajan et al. [25], Dorji et al. [26], Nakano [16], Das et al. [35], Maulana et al. [113], Saha et al. [27], and Iqbal et al. [20]. Host plant associations remain unknown for 104 species, representing approximately 59% of the members of the tribe Epilachnini recorded in China. This substantial lack of host plant information highlights a significant gap in our understanding of feeding habits and host plant interactions within this tribe. Host plant associations are essential for our understanding the evolutionary history of phytophagous lineages, including patterns of host shifts and host-associated diversification. Such data are also critical for predicting pest host ranges and developing effective management strategies.

## 3. Checklist

**Genus *Afidenta* Dieke, 1947**

*Afidenta* Dieke, 1947 [114]: 109. Type species: *Afidenta mimetica* Dieke, 1947 [114] (=*Epilachna misera* Weise, 1901a [115]: 420). Original designation. — Li & Cook [21]; 84; Liu [116]: 26; Pang & Mao [53]: 118; Fürsch [80]: 222; Chakraborty & Biswas [117]: 149; Jadwiszczak & Węgrzynowicz [1]: 15; Poorani [93]: 37, 38; Kovář [94]: 625; Ren et al. [9]: 250; Szawaryn et al. [34]: 558, 565; Tomaszewska & Szawaryn [2]: 56; Nakano [16]: 132; Bajracharya & Budha [99]: 848; Iqbal et al. [20]: 77.

**Distribution.** Palaearctic and Oriental regions [2].

**Remarks.** *Afidenta* currently includes only the type species, *Afidenta misera*, which has a notably broader distribution, extending across the Oriental region and into the Palaearctic (Shandong and Tibet) region. In China, it is widely distributed in Anhui, Fujian, Guangdong, Guangxi, Guizhou, Shandong, Taiwan, Yunnan, and Tibet. Host plant records include five plant families and ten species.

1) ***Afidenta misera* (Weise, 1901)**

*Epilachna misera* Weise, 1901a [115]: 420. Type locality: Sri Lanka.

*Afidenta mimetica* Dieke, 1947 [114]: 110. Type locality: Vietnam. — Li & Cook [21]; 67; Yu & Pang [118]: 14; Wu [119]: 5; Chakraborty & Biswas [117]: 150. Synonymized by Bielawski [120]: 392.

*Afidenta mimetica simplex* Dieke, 1947 [114]: 111. Type locality: India. Synonymized with *Afidenta mimetica* by Li & Cook [21]; 67.

*Afidenta misera* (Weise [115]): — Bielawski [120]: 392; Liu [116]: 26; Pang & Mao [53]: 119; Li [121]: 401; Miyatake [89]: 25; Xiao & Li [57]: 384; Wang & Cao [122]; 118; Yu & Pang [118]: 14; Katakura et al. [23]: 331; Islam et al. [123]: 127; Pang & Zeng [67]: 279; Jadwiszczak & Węgrzynowicz [1]: 19; Poorani [93]: 38; Kovář [94]: 625; Ren et al. [9]: 250; Zhang & Ou [109]: 57; Canepari [15]: 365; Katoh et al. [22]: 822; Szawaryn et al. [34]: 558, 565; Tomaszewska & Szawaryn [2]: 59; Nakano [16]: 132; Bajracharya & Budha [99]: 848; Iqbal et al. [20]: 79.

**Distribution. Oriental.** China (Anhui, Fujian, Guangdong, Guangxi, Guizhou, Taiwan, Yunnan); - Bangladesh, India, Indonesia, Myanmar, Nepal, Pakistan, Philippines, Sri Lanka, Thailand, Vietnam; **Palaearctic.** China (Shandong, Tibet) [1,2,9,20,73,89,93,99,106,114,115,123,124,125].
**Host plants. Aristolochiaceae** [22]; **Bignoniaceae:** *Catalpa fargesii* f. *duclouxii* [16]; **Ebenaceae:** *Diospyros strigosa* [16]; **Fabaceae:** *Desmodium* sp. [23], *Glycine max* [106,109], *G. soja* [16], *Rhynchosia minima*, *R. volubilis* [16,109], *Phaseolus vulgaris* [106], *Vigna unguiculata* [16,109]; **Solanaceae:** *Solanum nigrum* [20].
**Genus *Afidentula* Kapur, 1958**

*Afidentula* Kapur, 1958 [82]: 324. Type species: *Epilachna manderstjernae* Mulsant, 1853 [37]: 256. Original designation. – Pang & Mao [53]: 120; Chakraborty & Biswas [117]: 150; Jadwiszczak & Węgrzynowicz [1]: 22; Poorani [93]: 38; Kovár [94]: 625; Ren et al. [9]: 252; Tomaszewska & Szawaryn [11]: 27; Szawaryn et al. [34]: 559, 564; Tomaszewska & Szawaryn [2]: 79; Nakano [16]: 133; Bajracharya & Budha [99]: 848; Iqbal et al. [20]: 70; Ashfaque et al. [126]: 499.

**Remarks.** Originally, *Afidentula* comprised only nine species from Asia [11]. Szawaryn et al. [34] has found that four studied African species of *Afidenta* formed a monophyletic group with *Afidentula* and that probably all African species formerly classified in *Afidenta* may belong to *Afidentula*. Some other species of former *Epilachna* and *Henosepilachna* may also belong to this genus, such as *E. blaesa* and *H. acervata*. This genus has more than 47 valid species. The Chinese fauna comprises ten species, four of which are endemic. Three of these endemic species are known only from Yunnan, and one species from Guizhou and Sichuan. Provisional regional distribution records are as follows: Yunnan (7), Guangdong (4), Guangxi (4), Sichuan (4), Guizhou (2), Hong Kong (1), and Tibet (1); the exact locality of one species is unknown. Host plants are known to comprise five species, revealing associations with thirteen plant species under five families. Poaceae is the most frequent host family (8 species), followed by Lamiaceae (2), while Fabaceae, Verbenaceae, and Cucurbitaceae are each represented by a single species.

2) ***Afidentula bisquadripunctata* (Gyllenhal, 1808)**

*Coccinella bis quadripunctata* Gyllenhal *in* Schönherr, 1808 [127]: 186. Type locality: “India Orientali”.

*Epilachna herbigrada* Mulsant, 1850 [128]: 805. Type locality: “environs de Pondichéry”. Synonymized by Crotch, 1874 [38]: 89. — Weise [115]: 421; Dieke [114]: 112 (as *Afidenta bisquadripunctata*); Bielawski [129]: 78 (as *Afidenta herbigrada*); Jadwiszczak & Węgrzynowicz [1]: 22 (as *Afidentula herbigrada*); Poorani [93]: 38.

*Afissula bisquadripunctata* (Gyllenhal [127]): — Wang & Cao [122]: 118; Yu & Lau [108]: 171; Zeng [130]: 50; Ren et al. [9]: 254.

*Afidentula bisquadripunctata* (Gyllenhal [127]): — Pang & Mao [53]: 121; Cao [131]: 12; Canepari [132]; 57; Jadwiszczak & Węgrzynowicz [1]: 22; Poorani [93]: 38; Kovár [94]: 625; Zhang & Ou [109]: 57; Tomaszewska & Szawaryn [11]: 29; Hayat & Khan [133]: 348; Tomaszewska & Szawaryn [2]: 82; Nakano [16]: 133; Bajracharya & Budha [99]: 848; Iqbal et al. [20]: 72; Ashfaque et al. [126]: 499.

**Distribution. Oriental.** China (Guangdong, Guizhou, Guangxi, Yunnan, Hong Kong); - India, Nepal, Pakistan, Sri Lanka, Vietnam [1,11,20,99,126,134].
**Host plants. Poaceae:***Apluda mutica*, *Arthraxon hispidulus* [16,109].

3) ***Afidentula decimaculata* Cao & Wang, 1992**

*Afidentula decimaculata* Cao & Wang *in* Wang & Cao, 1992 [90]: 203, 205. Type locality: China (Yunnan). — Cao [131]: 12; Jadwiszczak & Węgrzynowicz [1]: 23; Kovár [94]: 625; Nakano [16]: 133; Tomaszewska & Szawaryn [11]: 49.

**Distribution. Oriental**. China (Yunnan) [11,90].
**Host plants. Lamiaceae:** *Colquhounia coccinea* [16,90], *Colquhounia* sp. [16].

4) ***Afidentula dentata* Wang, Tomaszewska & Ren, 2015**

*Afidentula dentata* Wang, Tomaszewska & Ren *in* Wang et al. 2015 [125]: 42. Type locality: China (Yunnan). —Nakano [16]: 133.

**Distribution. Oriental.** China (Yunnan) [125,134].

5) ***Afidentula himalayana* Kapur, 1963**

*Afidentula himalayana* Kapur, 1963 [49]: 12. Type locality: India. — Pang & Mao [52]: 324; Pang & Mao [53]: 123; Li [121]: 401; Canepari [135]: 30; Cao [131]: 12; Canepari [132]: 57; Jadwiszczak & Węgrzynowicz [1]: 23; Poorani [93]: 38; Kovár [94]: 625; Ren et al. [9]: 252; Canepari [15]: 365; Tomaszewska & Szawaryn [11]: 32; Tomaszewska & Szawaryn [2]: 82; Nakano [16]: 134; Dorji et al. [26]: 513; Bajracharya & Budha [99]: 848.

*Afidentula himalayana* var. *championi* Kapur, 1963 [49]: 16. Type locality: India. Synonymised by Jadwiszczak & Węgrzynowicz [1]: 23. Infrasubspecific name published after 1960, unavailable in zoological nomenclature.

**Distribution. Oriental.** Bhutan, India, Nepal; **Palaearctic**. China (Tibet) [1,11,134].
**Host plant**. **Poaceae** [16].

6) ***Afidentula jinpingensis* Wang, Tomaszewska & Ren, 2015**

*Afidentula jinpingensis* Wang, Tomaszewska & Ren in Wang et al. 2015 [125]: 44. Type locality: China (Yunnan). —Nakano [16]: 134.

**Distribution. Oriental**. China (Yunnan) [125,134].

7) ***Afidentula manderstjernae bielawskii* Tomaszewska & Szawaryn, 2013**

*Afidentula manderstjernae bielawskii* Tomaszewska & Szawaryn, 2013 [11]: 37. Type locality: Vietnam. — Katoh et al. [22]: 822.

**Distribution. Oriental.** China (locality not available); –Vietnam [11].
**Host plant. Poaceae**: *Microstegium ciliatum* [109].

8) ***Afidentula manderstjernae manderstjernae* (Mulsant, 1853)**

*Epilachna manderstjernae* Mulsant, 1853 [37]: 256. Type locality: Asia.

*Afidentula manderstjernae* (Mulsant [37]): — Kapur [82]: 325; Pang & Mao [53]: 122; Xiao & Li [57]: 384; Cao [131]: 12; Zeng [130]: 49; Canepari [132]: 58; Chakraborty & Biswas [117]: 150; Jadwiszczak & Węgrzynowicz [1]: 23; Canepari [136]: 265; Poorani [93]: 38; Kovár [94]: 625; Ren et al. [9]: 252; Zhang & Ou [109]: 57; Tomaszewska & Szawaryn [11]: 34; Saeed et al. [137]: 1367; Tomaszewska & Szawaryn [2]: 82; Nakano [16]: 134; Dorji et al. [26]: 513; Bajracharya & Budha [99]: 848; Iqbal et al. [20]: 72; Ashfaque et al. [126]: 500.

*Afidentula manderstgerne*: — an incorrect species name used by Saeed et al. [137]: 1371.

**Distribution. Oriental.** China (Sichuan, Guangdong, Guangxi, Yunnan); - India, Myanmar, Nepal, Pakistan, Vietnam [20,99,126].
**Host plants. Fabaceae:***Vigna radiata* [20]; **Poaceae:** *Heteropogon contortus*, *Zea mays* [20], *Microstegium ciliatum* [16,109]; **Verbenaceae:** *Lantana camara* [20].

9) ***Afidentula quindecemguttata* (Dieke, 1947)**

*Afissa quindecemguttata* Dieke, 1947 [114]: 126. Type locality: China (Sichuan).

*Afidentula quinquedecemguttata* Pang & Mao [53]: 122. Unnecessary emendation by Pang & Mao [53]: — Liu [116]: 30; Jadwiszczak & Węgrzynowicz [1]: 24.

*Afidentula quinquedecemguttata* (Dieke [114]): — Pang & Mao [53]: 122; Wang & Cao [122]: 121; Cao [131]: 12; Ren et al. [9]: 252; Zhang & Ou [109]: 57.

*Afidentula quindecemguttata* (Dieke [114]): — Jadwiszczak & Węgrzynowicz [1]: 24; Kovár [94]: 625; Tomaszewska & Szawaryn [11]: 41; Tomaszewska & Szawaryn [2]: 82; Nakano [16]: 134.

**Distribution. Oriental**. China (Guizhou, Sichuan) [1,125,134].
**Host plants. Cucurbitaceae:** *Gynostemma pentaphyllum* [16]; **Poaceae:** *Arthroxon hispidus* [16], *Phyllostachys pubescens*, *Yushania niitakayamensis* [16,109].

10) ***Afidentula siamensis* (Dieke, 1947)**

*Afissa siamensis* Dieke, 1947 [114]: 127. Type locality: Thailand.

*Afidenta siamensis* (Dieke [114]): — Pang & Mao [53]: 119; Jadwiszczak & Węgrzynowicz [1]: 21; Kovár [94]: 625; Ren et al. [9]: 250; Nakano [16]: 135.

*Afidentula siamensis* (Dieke [114]): — Nakano [16]: 135; Wang et al. [125]: 41.

**Distribution. Oriental.** China (Guizhou, Yunnan); –Thailand [1,125,134].

11) ***Afidentula thanhsonensis* Hoàng, 1977**

*Afidentula thanhsonensis* Hoàng, 1977 [87]: 134. Type locality: Vietnam (Vinh Phu, Than Shon). —Cao [131]: 12; Jadwiszczak & Węgrzynowicz [1]: 24; Nakano [16]: 135.

*Afidentula cucphuongensis* Hoàng, 1977 [87]: 135. Type locality: Vietnam (Xanamninh, Cuc Phuong). — Cao [131]: 12; Jadwiszczak & Węgrzynowicz [1]: 22. Synonymized by Tomaszewska & Szawaryn [11]: 46.

**Distribution. Oriental.** China (Guangdong); - India, Vietnam [1].
**Genus *Afissa* Dieke, 1947**

*Afissa* Dieke, 1947 [114]: 113. Type species: *Coccinella flavicollis* Thunberg, 1781 [138]: 18. Original designation. — Liu [116]: 26. ***Synonymised*** with *Epilachna* by Li & Cook [21]: 51. — Pang & Mao [53]: 130; Kovář [94]: 626; Ślipiński [31]: 535; Jadwiszczak & Węgrzynowicz [1]: 31; Poorani [93]: 40; Pang et al. [69]: 34. ***Resurrected*** from synonymy by Szawaryn et al. [34]: 556. — Tomaszewska & Szawaryn [2]: 49; Das et al. [35]: 38; Das et al. [139]: 249; Bajracharya & Budha [99]: 848; Iqbal et al. [20]: 61; Wang et al. [73]: 3.

*Afissula* Kapur, 1958 [82]: 319. Type species: *Afissula rana* Kapur, 1958 [82]: 320. Original designation. —Pang & Mao [53]: 124; Chakraborty & Biswas [117]: 150; Poorani [93]: 39. Synonymized by Szawaryn et al. [34]: 565.

**Remarks.** The genus *Afissa* currently includes 47 valid species, predominantly found in the Oriental region (44 species). An additional twelve species are included when extending the range into the Palaearctic region, with two species recently described from the Palaearctic and one species occurring in both the Oriental and Australasia regions. The Chinese fauna consists of 34 species, 16 of which are endemic to China. Provisional regional distribution records are as follows: Yunnan (15 species), Tibet (12), Guangxi (9), Taiwan (9), Guizhou (8), Sichuan (6), Henan (4), Hubei (4), Shaanxi (4), Anhui (3), Hainan (3), Hunan (3), Fujian (2), Guangdong (2), Hong Kong (2), Jiangxi (2), and 1 species each in Gansu, Jiangsu, and Zhejiang.

Among the endemic species, thirteen species are known from only one to three provinces, while three species have wider distribution. Host plant records are available for 14 species, revealing associations with 26 plant species from 11 families. Urticaceae is the most frequently recorded host family (9 species), followed by Rubiaceae (5), Asteraceae (3), and Lamiaceae (3). The families Araceae, Hydrangeaceae, Euphorbiaceae, Rosaceae, Schizaeaceae, and Styracaceae are each represented by a single species.

12) ***Afissa ampliata* (Pang & Mao, 1979)**

*Epilachna ampliata* Pang & Mao, 1979 [53]: 152. Type locality: China. — Wang & Cao [91]; 120; Wang & Cao [122]: 118; Jadwiszczak & Węgrzynowicz [1]: 35; Kovář [94]: 626; Ren et al. [9]: 262, 263; Canepari [15]: 366; Pang et al. [69]: 34.

*Afissa ampliata* (Pang & Mao [53]): — Tomaszewska & Szawaryn [2]: 52; Nakano [16]: 139; Poorani & Thangjam [140]: 551; Bajracharya & Budha [99]: 849.

**Distribution. Oriental.** China (Guangxi, Yunnan, Guizhou); - India, Nepal [2,53,73,99,140].

13) ***Afissa anhweiana* Dieke, 1947**

*Afissa anhweiana* Dieke, 1947 [114]: 147. Type locality: China (Anhui). — Tomaszewska & Szawaryn [2]: 52; Nakano [16]: 140.

*Epilachna anhweiana* (Dieke [114]): — Wang & Cao [91]: 120; Jadwiszczak & Węgrzynowicz [1]: 36; Kovář [94]: 626; Ren et al. [9]: 264; Pang et al. [69]: 4, 34.

**Distribution. Oriental.** China (Anhui, Guangdong, Guangxi, Guizhou, Henan, Hubei, Hunan, Jiangsu, Jiangxi, Yunnan, Zhejiang); - **Palaearctic.** China (Shaanxi) [1,69,114,134].
**Host plant. Styracaceae:** *Styrax japonica* [16].

14) ***Afissa arisana* (Li & Cook, 1961)**

*Afidenta arisana* Li & Cook, 1961 [21]: 87. Type locality: China (Taiwan). — Yu & Pang [118]: 14; Jadwiszczak & Węgrzynowicz [1]: 15; Nakano [16]: 135.

*Afissula arisana* (Li & Cook [21]): — Ren et al. [9]: 254.

*Afissa arisana* (Li & Cook [21]): — Tomaszewska & Szawaryn [2]: 53.

**Distribution. Oriental**. China (Guangxi, Taiwan) [1,16,21].
**Host plant. Araceae**: *Alocasia macrorrhizos* [16].

15) ***Afissa chinensis* (Weise, 1912)**

*Solanophila chinensis* Weise, 1912a [43]: 112. Type locality: China (Fujian).

*Afissa chinensis* var. *separata* Dieke, 1947 [114]: 150. Type locality: China (Anhui). Synonymized by Li & Cook [21]: 74.

*Afissa chinensis tsushimana* Nakane & Araki, 1960 [141]: A118. Type locality: Japan. — Katoh et al. [22]: 822.

*Epilachna chinensis* (Weise [43]): — Li & Cook [21]; 74; Pang & Mao [53]: 154; Zhang & Qi [142]: 249; Xiao & Li [57]: 385; Pang [58]: 108, 109; Yu & Pang [118]: 14; Zheng & Pang [143]: 118; Kuznetsov & Zakharov [144]: 173; Jadwiszczak & Węgrzynowicz [1]: 48; Kovář [94]: 627; Ren et al. [9]: 268; Pang et al. [69]: 6; Cho [145]: 311.

*Afissa chinensis* (Weise [43]): — Nakano [16]: 142.

**Distribution. Oriental.** China (Anhui, Fuijan, Guangdong, Hainan, Hubei, Guangxi, Guizhou, Jiangxi, Yunnan, Taiwan); **Palaearctic.** China (Henan, Shaanxi); - Japan, Russian Far East [58,69,141,144].
**Host plants. Rubiaceae:** *Galium aparine*, *G. spurium, Paederia scandens*, *Rubia akane*, *R. cordifolia*; **Schizaeaceae:** *Lygodium japonicum*; **Urticaceae:** *Boehmeria* sp. [16].

16) ***Afissa craspedotricha* (Yu, 2004)**

*Afissula craspedotricha* Yu, 2004 [68]: 60. Type locality: China (Tibet). — Ren et al. [9]: 254; Nakano [16]: 135.

*Afissa craspedotricha* (Yu [68]): — Poorani & Thangjam [140]: 552.

**Distribution. Oriental**. India; **Palaearctic**. China (Tibet) [68,140].

17) ***Afissa cuonaensis* (Pang & Mao, 1977)**

*Epilachna cuonaensis* Pang & Mao, 1977 [52]: 324, 325. Type locality: China (Tibet). — Li [121]: 400; Jadwiszczak & Węgrzynowicz [1]: 55; Kovář [94]: 627; Ren et al. [9]: 270; Pang et al. [69]: 34; Nakano [16]: 144.

*Afissa cuonaensis* (Pang & Mao [52]): — Iqbal et al. [20]: 62.

**Distribution. Oriental**. India, Pakistan; **Palaearctic**. China (Tibet) [20,52,69].

18) ***Afissa dumerili* (Mulsant, 1850)**

*Epilachna dumerili* Mulsant, 1850 [128]: 801. Type locality: “Indes Orientales”. — Miyatake [89]: 25; Canepari [135]: 29; Jadwiszczak & Węgrzynowicz [1]: 60; Poorani [93]: 41; Kovář [94]: 627; Ren et al. [9]: 272; Zhang & Ou [109]: 53; Canepari [15]: 365; Pang et al. [69]: 35; Dorji et al. [26]: 515; Nakano [16]: 145.

*Solanophila dumerili* a. *discordia* Mader, 1935 [146]: 362. — Jadwiszczak & Węgrzynowicz [1]: 60; Poorani [93]: 41.

*Afissa dumerili* (Mulsant [128]): — Kapur [85]: 159; Canepari [132]: 59; Sajan et al. [25]: 1243; Bajracharya & Budha [99]: 849.

**Distribution. Oriental.** China (Fujian, Taiwan, Hong Kong, Yunnan); - Bhutan, Myanmar, Nepal, Sri Lanka, Vietnam [69,93,99].
**Host plants. Lamiaceae:** *Clerodendrum infortunatum*, *Colebrookea oppositifolia* [25].

19) ***Afissa expansa* Dieke, 1947**

*Afissa expansa* Dieke, 1947 [114]: 141. Type locality: China (Sichuan). — Tomaszewska & Szawaryn [2]: 52.

*Afissula expansa* (Dieke [114]): — Pang & Mao [53]: 127; Wang & Cao [92]: 1; Wang & Cao [122]: 118; Jadwiszczak & Węgrzynowicz [1]: 26; Kovář [94]: 626; Ren et al. [9]: 256; Zhang & Ou [109]: 57; Nakano [16]: 136.

**Distribution. Oriental.** China (Yunnan, Sichuan, Hunan, Hubei, Hainan, Guizhou); **Palaearctic.** China (Henan) [1,114,134].
**Host plants. Urticaceae:** *Elatostema* sp. [16], *Urtica fissa* [16,109], *U. thunbergiiana* [16].

20) ***Afissa flavicollis* (Thunberg, 1781)**

*Coccinella flavicollis* Thunberg, 1781 [138]: 18. Type locality: East Indies.

*Epilachna flavicollis* (Thunberg [138]): — Chakraborty & Biswas [117]: 145; Jadwiszczak & Węgrzynowicz [1]: 65; Poorani [93]: 41; Pang et al. [69]: 35.

*Afissa flavicollis* (Thunberg [138]): — Tomaszewska & Szawaryn [2]: 52.

*Epilachna fiavicollis* (Thunberg [138]): — an incorrect spelling used by Nakano [16]: 146.

**Distribution. Oriental.** China (Taiwan); - Myanmar, India, Sri Lanka, Thailand, Vietnam, Philippines, Indonesia (Sumatra, Java, Borneo, Celebes) [1,69].

21) ***Afissa galerucinoides* (Korschefsky, 1934)**

*Epilachna* (*Solanophila*) *galerucinoides* Korschefsky, 1934a [147]: 107. Type locality: Indonesia.

*Epilachna galerucinoides* Korschefsky [147]: — Bielawski [148]: 193; Pang & Mao [53]: 155; Li [149]: 222; Wang & Cao [91]; 120; Wang & Cao [122]: 117; Jadwiszczak & Węgrzynowicz [1]: 68; Kovář [94]: 626; Ślipiński [31]: 542; Ren et al. [9]: 276; Pang et al. [69]: 7, 35; Nakano [16]: 146.

*Afissa galerucinoides* (Korschefsky [147]): — Szawaryn [150]: 161.

**Distribution. Australasia.** Australia; **Oriental.** China (Anhui, Guangxi, Hainan, Yunnan, Guizhou, Taiwan), Vietnam, Thailand, Sri Lanka, Indonesia [1,16,69,150].

22) ***Afissa gedeensis* Dieke, 1947**

*Afissa gedeensis* Dieke, 1947 [114]: 129. Type locality: Indonesia (Java). — Tomaszewska & Szawaryn [2]: 52; Nakano [16]: 147; Maulana et al. [113]: 56.

*Epilachna gedeensis* (Dieke [114]): — Wang & Cao [91]: 120; Wang & Cao [122]: 117; Katakura et al. [23]: 331; Jadwiszczak & Węgrzynowicz [1]: 68; Kovář [94]: 626; Ren et al. [9]: 276; Pang et al. [69]: 35; Katoh et al. [22]: 822.

**Distribution. Oriental.** China (Guizhou, Hunan, Sichuan, Yunnan); - Indonesia (Java) [2,69,114].
**Host plants. Asteraceae:** *Ageratina* sp. [16]; **Urticaceae** [22], *Elatostema* sp. [113], *E. acuminata* [16,23], *E. strigosum* [23].

23) ***Afissa gibbera* (Crotch, 1874)**

*Epilachna gibbera* Crotch, 1874 [38]: 80. Type locality: India. — Canepari [135]: 29; Chakraborty & Biswas [117]: 145; Jadwiszczak & Węgrzynowicz [1]: 69; Poorani [93]: 41; Nakano [16]: 147.

*Afissa gibbera* (Crotch [38]): — Kapur [49]: 4; Das et al. [35]: 249; Bajracharya & Budha [99]: 849.

**Distribution. Oriental**. Nepal, India; **Palaearctic**. China (Tibet) [1,16].

24) ***Afissa henanica* (Yu, 2000)**

*Afissula henanica* Yu, 2000 [63]: 68. Type locality: China (Henan). — Kovář [94]: 626; Nakano [16]: 136; Ren et al. [9]: 256.

*Afissa henanica* (Yu [63]): — Tomaszewska & Szawaryn [2]: 53.

**Distribution. Palaearctic**. China (Henan, Shaanxi) [63,134].

25) ***Afissa hydrangeae* (Pang & Mao, 1979)**

*Afissula hydrangeae* Pang & Mao, 1979 [53]: 125. Type locality: China (Sichuan). — Pang & Mao [53]: 125; Jadwiszczak & Węgrzynowicz [1]: 26; Kovář [94]: 626; Nakano [16]: 136; Ren et al. [9]: 256.

*Afissa hydrangeae* (Pang & Mao [53]): — Tomaszewska & Szawaryn [2]: 53.

**Distribution. Oriental.** China (Sichuan) [1,53].
**Host plant. Hydrangeaceae**: *Hydrangea* sp. [16].

26) ***Afissa incauta* (Mulsant, 1850)**

*Epilachna incauta* Mulsant, 1850 [128]: 803. Type locality: Indonesia (Java). — Li & Cook [21]: 65; Yu & Pang [118]: 15; Katakura et al. [23]: 331; Jadwiszczak & Węgrzynowicz [1]: 76; Poorani [93]: 42; Kovář [94]: 627; Pang et al. [69]: 35; Katoh et al. [22]: 822; Nakano [16]: 149.

*Afissa incauta* (Mulsant [128]): — Maulana et al. [113]: 57.

**Distribution. Oriental.** China (Taiwan); - Indonesia (Java, Sumatra), Myanmar [1,69].
**Host plants. Urticaceae** [22], *Boehmeria clidemioides* [113], *B. macrophylla*, *Debregeasia wallichiana* [23], *Elatostema* sp. [113], *Leucosyke candidissima*, *Villebrunea* sp. [23].

27) ***Afissa kambaitana* (Bielawski, 1967)**

*Epilachna kambaitana* Bielawski, 1967a [151]: 793. Type locality: Myanmar.

*Afissula kambaitana* (Bielawski [151]): — Li [121]: 402; Canepari [132]: 63; Pang & Mao [52]: 324; Pang & Mao [53]: 127; Jadwiszczak & Węgrzynowicz [1]: 26; Poorani [93]: 39; Kovář [94]: 626; Nakano [16]: 136.

*Afissa kambaitana* (Bielawski [151]): — Bajracharya & Budha [99]: 849.

**Distribution. Oriental.** Myanmar, Nepal; **Palaearctic**. China (Tibet) [1,99,134].

28) ***Afissa langpingensis* (Zeng & Yang, 1996)**

*Epilachna langpingensis* Zeng & Yang, 1996b [60]: 195, 200. Type locality: China (Guangxi). —Jadwiszczak & Węgrzynowicz [1]: 82; Kovář [94]: 628; Ren et al. [9]: 282; Pang et al. [69]: 35; Nakano [16]: 150.

*Epilachna longpingensis* Zeng & Yang [60]: — an incorrect spelling used by Nakano [16]: 150.

*Afissa langpingensis* (Zeng & Yang [60]): — Das et al. [36]: 79.

**Distribution. Oriental.** China (Guangxi) [60].

29) ***Afissa max* (Pang & Ślipiński, 2012)**

*Epilachna max* Pang & Ślipiński *in* Pang et al. [69]: 26. Type locality: China (Sichuan). —Nakano [16]: 151.

*Afissa max* (Pang & Ślipiński *in* Pang et al. [69]): — Tomaszewska & Szawaryn [2]: 52.

**Distribution. Oriental.** China (Hubei, Sichuan, Taiwan) [2,69,73].

30) ***Afissa motuoensis* Jin, Chen & Wang, 2026**

*Afissa motuoensis* Jin, Chen & Wang *in* Jin et al. 2026 [74]: 307. Type locality: China (Xizang).

**Distribution. Palaearctic.** China (Tibet) [74].

31) ***Afissa mystica* (Mulsant, 1850)**

*Epilachna mystica* Mulsant, 1850 [128]: 841. Type locality: India. — Pang & Mao [52]: 324; Li [121]: 399; Booth & Pope [152]: 346; Chakraborty & Biswas [117]: 147; Jadwiszczak & Węgrzynowicz [1]: 94; Poorani [93]: 43; Kovář [94]: 628; Ren et al. [9]: 290; Canepari [15]: 367; Pang et al. [69]: 35; Dorji et al. [26]: 515; Nakano [16]: 154; Bajracharya & Budha [99]: 852.

*Afissa mystica* (Mulsant [128]): — Kapur [82]: 315; Das et al. [35]: 38.

**Distribution. Oriental.** Bhutan, India, Nepal, Myanmar; **Palaearctic.** China (Tibet) [1,69,99].

32) ***Afissa mysticoides* (Sicard, 1913)**

*Solanophila mysticoides* Sicard, 1913 [153]: 507. Type locality: India.

*Epilachna mysticoides* (Sicard [153]): — Li [121]: 399.

*Afissula mysticoides* (Sicard [153]): — Pang & Mao [53]: 129; Miyatake [89]: 25; Canepari [136]: 265; Chakraborty & Biswas [117]: 147; Jadwiszczak & Węgrzynowicz [1]: 26; Poorani [93]: 39; Kovář [94]: 626; Canepari [15]: 367; Nakano [16]: 136; Ren et al. [9]: 258; Dorji et al. [26]: 514.

*Afissa mysticoides* (Sicard [153]): — Kapur [82]: 316; Kapur [84]: 291; Canepari [132]: 61; Bajracharya & Budha [99]: 849.

**Distribution. Oriental**. Bhutan, India, Nepal; **Palaearctic**. China (Tibet) [1,89,99,134].

33) ***Afissa nielamuensis* (Pang & Mao, 1977)**

*Epilachna nielamuensis* Pang & Mao, 1977 [52]: 323, 327. Type locality: China (Tibet). — Li [121]: 398; Miyatake [89]: 30; Jadwiszczak & Węgrzynowicz [1]: 94; Poorani [93]: 44; Kovář [94]: 628; Ren et al. [9]: 290; Pang et al. [69]: 35; Nakano [16]: 154.

*Afissa nielamuensis* (Pang & Mao [52]): — Canepari [132]: 59; Poorani & Thangjam [140]: 553; Das et al. [35]: 38; Bajracharya & Budha [99]: 849.

**Distribution. Oriental**. China (Yunnan); - India, Nepal; **Palaearctic**. China (Tibet) [52,69,89,99,140].

34) ***Afissa nonggangensis* (Zeng & Yang, 1996)**

*Afissula nonggangensis* Zeng & Yang, 1996a [59]: 271, 274. Type locality: China (Guangxi). — Jadwiszczak & Węgrzynowicz [1]: 26; Kovář [94]: 626; Nakano [16]: 137.

*Afissula longgangensis* Zeng, 1996 [59]. — an incorrect spelling used by Ren et al. [9]: 258.

*Afissa longgangensis* (Zeng & Yang [59]): — Tomaszewska & Szawaryn [2]: 53.

**Distribution. Oriental.** China (Guangxi) [1,59,134].
**Host plant. Euphorbiaceae**: *Claoxylon khasianum* [16,59].

35) ***Afissa ornithorrhyncha* (Zeng & Yang, 1996)**

*Afissula ornithorrhyncha* Zeng & Yang, 1996a [59]: 273, 275. Type locality: China (Guangxi). — Jadwiszczak & Węgrzynowicz [1]: 25; Kovář [94]: 626; Nakano [16]: 137.

*Afissula ornithorrhynchus* Zeng [59]: — an incorrect spelling used by Ren et al. [9]: 258; Zhang & Ou [109]: 57.

*Afissa ornithorrhyncha* (Zeng & Yang [59]): — Tomaszewska & Szawaryn [2]: 53.

**Distribution. Oriental**. China (Guangxi, Yunnan) [1,59,134].
**Host plant. Rosaceae**: *Neillia sinensis* [16,109].

36) ***Afissa plicata* (Weise, 1889)**

*Epilachna plicata* Weise, 1889 [39]: 649. Type locality: China (Gansu). — Pang & Mao [53]: 151; Wang & Cao [91]: 120; Wang & Cao [92]: 1; Jadwiszczak & Węgrzynowicz [1]: 106; Kovář [94]: 628; Ren et al. [9]: 294; Pang et al. [69]: 36.

*Afissa plicata* (Weise [39]): — Liu [116]: 230; Tomaszewska & Szawaryn [2]: 53; Nakano [16]: 156.

**Distribution. Oriental.** China (Taiwan, Yunnan, Sichuan, Guizhou); **Palaearctic.** China (Shaanxi, Gansu) [69].
**Host plants. Asteraceae:** *Artemisia rubripes, Tanacetum vulgare* [16].

37) ***Afissa puncta* (Bielawski, 1967)**

*Afissula puncta* Bielawski, 1967b [154]: 154. Type locality: Myanmar. — Jadwiszczak & Węgrzynowicz [1]: 27; Poorani [93]: 39.

*Afissa puncta* (Bielawski [154]): — Tomaszewska & Szawaryn [2]: 53.

**Distribution**. **Oriental**. China (Yunnan); - Myanmar [1,59,134].

38) ***Afissa pyramidalis* Jin, Chen & Wang, 2026**

*Afissa pyramidalis* Jin, Chen & Wang *in* Jin et al. 2026 [74]: 309. Type locality: China (Xizang).

**Distribution**. **Palaearctic**. China (Tibet) [74].

39) ***Afissa rana* (Kapur, 1958)**

*Afissula rana* Kapur, 1958 [82]: 320. Type locality: Nepal. — Pang & Mao [52]: 324; Pang & Mao [53]: 124; Li [121]: 401; Miyatake [89]: 25; Canepari [132]: 62; Jadwiszczak & Węgrzynowicz [1]: 27; Canepari [136]: 265; Poorani [93]: 39; Kovář [94]: 626; Zhang & Ou [109]: 57; Canepari [15]: 367; Nakano [16]: 137; Ren et al. [9]: 260.

*Afissa rana* (Kapur [82]): — Tomaszewska & Szawaryn [2]: 53; Sajan et al. [25]: 1244; Das et al. [35]: 38; Bajracharya & Budha [99]: 850.

**Distribution. Oriental**. India, Nepal; **Palaearctic**. China (Tibet) [1,99,134].
**Host plants. Urticaceae**: *Urtica* sp. [16,25], *U. fissa* [16].

40) ***Afissa sanscrita* (Crotch, 1874)**

*Epilachna sanscrita* Crotch, 1874 [38]: 82. Type locality: India.

*Afissula sanscrita* (Crotch [38]): — Kapur [49]: 4, 12; Pang & Mao [52]: 324; Pang & Mao [53]: 128; Li [121]: 402; Canepari [135]: 30; Canepari [132]: 61; Chakraborty & Biswas [117]: 151; Jadwiszczak & Węgrzynowicz [1]: 27; Poorani [93]: 39; Kovář [94]: 626; Nakano [16]: 137.

*Afissa sanscrita* (Crotch [38]): — Tomaszewska & Szawaryn [2]: 53; Bajracharya & Budha [99]: 850.

**Distribution**. **Oriental**. Nepal, India; **Palaearctic**. China (Tibet) [1,99].

41) ***Afissa spatulata* (Cao & Xiao, 1984)**

*Afissula spatulata* Cao & Xiao, 1984 [55]: 110, 126. Type locality: China (Yunnan). — Jadwiszczak & Węgrzynowicz [1]: 27; Kovář [94]: 626; Nakano [16]: 137.

**Distribution. Oriental**. China (Yunnan) [1,55,134].

42) ***Afissa undecimspilota* (Hope, 1831)**

*Coccinella 11-spilota* Hope, 1831 [155]: 31. Type locality: Nepal.

*Epilachna undecimspilota* (Hope [155]): — Pang & Mao [52]: 324; Chakraborty & Biswas [117]: 147; Jadwiszczak & Węgrzynowicz [1]: 125; Poorani [93]: 45; Kovář [94]: 629; Pang et al. [69]: 36; Dorji et al. [26]: 516; Nakano [16]: 159; Bajracharya & Budha [99]: 852.

*Afissa undecimspilota* (Hope [155]): — Das et al. [35]: 38.

**Distribution. Oriental.** China (Taiwan, Hong Kong); - Bhutan, Nepal, India, Myanmar, Indonesia (Sumatra), Sri Lanka, Thailand; **Palaearctic.** China (Tibet) [1,69,99].

43) ***Afissa uniformis* (Pang & Mao, 1979)**

*Afissula uniformis* Pang & Mao, 1979 [53]: 126. Type locality: China (Sichuan). — Xiao & Li [57]: 384; Jadwiszczak & Węgrzynowicz [1]: 28; Kovář [94]: 626; Nakano [16]: 138; Ren et al. [9]: 260.

*Afissa uniformis* (Pang & Mao [53]): — Tomaszewska & Szawaryn [2]: 53.

**Distribution. Oriental.** China (Guangxi, Sichuan, Yunnan) [53,134].
**Host plant. Vitaceae** [16].

44) ***Afissa xuexii* Wang & Wang, 2025**

*Afissa xuexii* Wang & Wang *in* Wang et al. 2025 [73]: 4. Type locality: China (Yunnan).

**Distribution. Oriental**. China (Guizhou, Yunnan) [73].

45) ***Afissa yunnanica* (Cao & Wang, 1992)**

*Afissula yunnanica* Cao & Wang, 1992 [56]: 66, 68. Type locality: China (Yunnan). — Jadwiszczak & Węgrzynowicz [1]: 28; Kovář [94]: 626; Nakano [16]: 138.

*Afissula yunanica* Cao & Wang [56]: — an incorrect spelling used by Ren et al. [9]: 260.

*Afissa yunnanica* (Cao & Wang [56]): — Tomaszewska & Szawaryn [2]: 53.

**Distribution. Oriental.** China (Yunnan) [1,56,134].
**Host plants. Lamiaceae**: *Elsholtzia ciliata* [16].
**Genus *Cynegetis* Chevrolat, 1837**

*Cynegetis* Chevrolat *in* Dejean, 1837 [156]: 461. Type species: *Coccinella impunctata* Linnaeus, 1767 [157]. Subsequent designation by Crotch [38]. — Fürsch [79]: 397; Jadwiszczak & Węgrzynowicz [1]: 193; Kovář [94]: 625; Bousquet & Bouchard, 2013 [158]: 140; Wang et al. [71]: 39; Szawaryn et al. [34]: 565; Tomaszewska & Szawaryn [2]: 62; Cho [145]: 311; Nakano [16]: 167.

**Remarks.** The genus *Cynegetis* includes three described species, all endemic to the Palaearctic region. *Cynegetis chinensis* is the only representative of the genus recorded in China; however, its host plant data remains unknown. Host plant data are available only for *Cynegetis impunctata*, which is associated with Poaceae.

46) ***Cynegetis chinensis* Wang & Ren, 2014**

*Cynegetis chinensis* Wang & Ren *in* Wang et al. 2014 [71]: 42. Type locality: China (Ningxia). — Tomaszewska & Szawaryn [2]: 65; Nakano [16]: 167.

**Distribution. Palaearctic**. China (Ningxia) [71].
**Genus *Diekeana* Tomaszewska & Szawaryn, 2015**

*Diekeana* Tomaszewska & Szawaryn *in* Szawaryn et al. [34]: 562. Type species: *Epilachna alternans* Mulsant, 1850 [128]: 767. Original designation. — Tomaszewska & Szawaryn [2]: 71; Das et al. [35]: 38; Chandra et al. [159]: 417; Jung et al. [160]: 241; Bajracharya & Budha [99]: 850.

**Remarks.** The genus *Diekeana* currently includes nineteen species, predominately found in the Oriental region, with four species extending into the Palaearctic. The Chinese fauna includes nine species, two of which are endemic: *Diekeana parainsignis* (distributed in Guangxi, Guizhou, and Yunnan) and *Diekeana bocaki* (known only from Sichuan). Provisional distribution records by regions are as follows: Guangxi (7), Yunnan (6), Guizhou (5), Sichuan (4), Guangdong (3), Fujian (3), Hunan (3), Taiwan (3), Anhui (2), Shaanxi (2), Hainan (3), Hubei (2), Jiangsu (2), Tibet (2), and 1 species each in Beijing, Gansu, Hebei, Henan, Jiangxi, and Zhejiang. Host plant records are available for eight of the nine species (all except *Diekeana bocaki*), revealing associations with twenty-one plant species under seven families. Cucurbitaceae is the most frequently recorded host family (8 spp.), followed by Solanaceae (6), Lamiaceae (3), and Urticaceae (2), while Asteraceae, Ranunculaceae, and Vitaceae are each represented by a single species.

47) ***Diekeana admirabilis* (Crotch, 1874)**

*Epilachna admirabilis* Crotch, 1874 [38]: 81. Type localities: China & Japan. — Li & Cook [21]: 56; Pang & Mao [53]: 136; Zhang & Qi [142]: 250; Xiao & Li [57]: 384; Pang [58]: 106; Wang & Cao [91]: 120; Wang & Cao [122]: 117; Yu & Pang [118]: 14; Kobayashi et al. [161]: 148; Zheng & Pang [143]: 118; Pang & Zeng [67]: 279; Jadwiszczak & Węgrzynowicz [1]: 32; Kovář [94]: 626; Ren et al. [9]: 262; Zhang & Ou [109]: 55; Pang et al. [69]: 2; Katoh et al. [22]: 822; Cho [145]: 311.

*Afissa admirabilis continentalis* Dieke, 1947 [114]: 118. Type locality: China (Sichuan). — Synonymized by Li & Cook [21]: 56; Liu [116]: 28; Jadwiszczak & Węgrzynowicz [1]: 32; Kovář [94]: 626.

*Epilachna admirabilis taiwanensis* Miyatake, 1965 [51]: 50. Type locality: China (Taiwan). — Jadwiszczak & Węgrzynowicz [1]: 32; Kovář [94]: 626.

*Afissa admirabilis* (Crotch [38]): — Liu [116]: 27.

*Diekeana admirabilis* (Crotch [38]): — Szawaryn et al. [34]: 562; Tomaszewska & Szawaryn [2]: 74; Nakano [16]: 138.

**Distribution. Oriental.** China (Anhui, Fujian, Hubei, Hunan, Guangxi, Guizhou, Jiangsu, Yunnan, Sichuan, Zhejiang, Taiwan); - Bangladesh, India, Myanmar, Nepal, Thailand, Vietnam; **Palaearctic.** China (Shaanxi); - Japan [1,2,58,69,134].
**Host plants. Cucurbitaceae** [22,161], *Benincasa hispida*, *Cucurbita moschata*, *Gynostemma pentaphyllum*, *Momordica charantia* [16,109], *Zehneria mucronata*; **Ranunculacea:** *Clematis petrae* [16]; **Solanaceae:** *Physalis alkekengi*, *Solanum melongena*, *S. nigrum*; **Vitaceae:** *Tetrastigma formosanum* [16,109].

48) ***Diekeana bocaki* (Pang & Ślipiński, 2012)**

*Epilachna bocaki* Pang & Ślipiński *in* Pang et al. 2012 [69]: 27. Type locality: China (Sichuan). — Nakano [16]: 141.

*Diekeana bocaki* (Pang & Ślipiński *in* Pang et al. 2012 [69]): — Szawaryn et al. [34]: 563; Ohta-Matsubayashi et al. [24]: 25.

**Distribution. Oriental**. China (Sichuan) [69].

49) ***Diekeana concuongensis concuongensis* (Hoàng, 1978)**

*Epilachna concuongensis* Hoàng, 1978 [88]: 837. Type locality: Vietnam. — Zhang & Pang [143]: 116, 119; Jadwiszczak & Węgrzynowicz [1]: 51; Kovář [94]: 626; Pang et al. [69]: 34; Nakano [16]: 143.

*Diekeana concuongensis* (Hoàng [88]): — Szawaryn et al. [34]: 563.

**Distribution. Oriental.** China (Guangxi); - Vietnam [1,24,69].
**Host plants. Cucurbitaceae**: *Sechium edule*, *Trichosanthes* sp. [24].

50) ***Diekeana glochinosa* (Pang & Mao, 1979)**

*Epilachna glochinosa* Pang & Mao, 1979 [53]: 138. Type locality: China (Yunnan). — Wang & Cao [91]: 120; Wang & Cao [122]: 117; Jadwiszczak & Węgrzynowicz [1]: 70; Kovář [94]: 626; Ren et al. [9]: 278; Zhang & Ou [109]: 55; Pang et al. [69]: 35.

*Epilachna glochiosa* Pang & Mao, 1979 [53]. An incorrect spelling by Szawaryn et al. [34]: 563.

*Diekeana glochinosa* (Pang & Mao [53]): — Szawaryn et al. [34]: 563; Tomaszewska & Szawaryn [2]: 74; Chandra et al. [159]: 417; Ohta-Matsubayashi et al. [24]: 25; Poorani & Thangjam [140]: 554; Nakano [16]: 147.

**Distribution. Oriental.** China (Guangxi, Hunan, Yunnan); - India (Nagaland) [69,134,140].
**Host plants. Urticaceae:***Urtica* sp. [24], *U. fissa* [16,109]; **Lamiaceae:** *Clerodendrum* sp. [24], *C. bungei* [16,109], *C. trichotomum* [16].

51) ***Diekeana grayi* (Mulsant, 1850)**

*Epilachna grayi* Mulsant, 1850 [128]: 774. Type locality: India. — Pang & Mao [52]: 324; Pang & Mao [53]: 138; Li [121]: 400; Miyatake [89]: 27; Booth & Pope [152]: 354; Yu & Pang [118]: 15; Chakraborty & Biswas [117]: 145; Jadwiszczak & Węgrzynowicz [1]: 72; Poorani [93]: 41; Kovář [94]: 627; Poorani [93]: 42; Canepari [15]: 366; Pang et al. [69]: 35; Nakano [16]: 148; Chandra et al. [159]: 417.

*Afissa grayi* (Mulsant [128]): — Kapur [82]: 324.

*Epilachna hopeiana* Miyatake, 1985 [89]: 28. Type locality: Nepal (Kathmandu). — Canepari [132]: 57; Jadwiszczak & Węgrzynowicz [1]: 75; Poorani [93]: 42; Kovář [94]: 627; Ren et al. [9]: 280; Pang et al. [69]: 35. Synonymized by Ohta-Matsubayashi et al. [24]: 22.

*Diekeana hopeiana* (Miyatake) — Szawaryn et al. [34]: 563; Nakano [16]: 149.

*Diekeana grayi* (Mulsant [128]): — Szawaryn et al. [34]: 562; Ohta-Matsubayashi et al. [24]: 22; *Bajracharya* & Budha [99]: 850.

**Distribution. Oriental.** China (Guangxi, Guizhou, Taiwan); - India, Indonesia (Java, Sumatra), Nepal, Vietnam; **Palaearctic.** China (Tibet) [1,69,134].
**Host plant. Cucurbitaceae:** *Sechium edule* [24].

52) ***Diekeana insignis* (Gorham, 1892)**

*Epilachna insignis* Gorham, 1892 [40]: 84. Type locality: China (Jiangxi). — Pang & Mao [53]: 132; Xiao & Li [57]: 385; Wang & Cao [91]: 120; Zhang & Yu [162]: 430; Zheng & Pang [143]: 118; Jadwiszczak & Węgrzynowicz [1]: 77; Poorani [93]: 42; Kovář [94]: 627; Ren et al. [9]: 282; Pang et al. [69]: 13, 35; Yu [163]: 188.

*Epilachna fairmairei* Frivaldszky, 1892 [41]. 121. Type locality: China (Guangdong). — Jadwiszczak & Węgrzynowicz [1]: 77; Kovář [94]: 627. Synonymized by Korschefsky [76].

*Afissa insignis* (Gorham [40]): — Liu [116]: 28.

*Diekeana insignis* (Gorham [40]): — Szawaryn et al. [34]: 562; Tomaszewska & Szawaryn [2]: 74; Nakano [16]: 149; Jung et al. [160]: 241.

**Distribution. Oriental.** China (Anhui, Hainan, Hubei, Fujian, Guangdong, Guangxi, Guizhou, Hainan, Jiangxi, Sichuan, Yunnan); - **Palaearctic.** China (Beijing, Hebei, Henan, Gansu, Jiangsu, Shaanxi, Shandong); - Japan, Korea (South) [69,160].
**Host plant. Solanaceae:** *Solanum melongena*, *S. nigrum* [16].

53) ***Diekeana macularis* (Mulsant, 1850)**

*Epilachna macularis* Mulsant, 1850 [128]: 797. Type locality: Nepal. — Pang & Mao [53]: 137; Booth & Pope [152]: 346; Xiao & Li [57]: 386; Wang & Cao [91]: 120; Wang & Cao [122]: 117; Chakraborty & Biswas [117]: 146; Jadwiszczak & Węgrzynowicz [1]: 86; Poorani [93]: 43; Kovář [94]: 628; Ren et al. [9]: 284; Zhang & Ou [109]: 55; Canepari [15]: 366; Pang et al. [69]: 13, 35.

*Solanophila macularis* ab. *donckieri* Weise, 1912a [43]: 112. Type locality: China (Yunnan). — Jadwiszczak & Węgrzynowicz [1]: 86; Poorani [93].

*Diekeana macularis* (Mulsant [128]): — Szawaryn et al. [34]: 562; Tomaszewska & Szawaryn [2]: 74; Nakano [16]: 150; Das et al. [35]: 38; Bajracharya & Budha [99]: 850.

**Distribution. Oriental.** China (Fujian, Guangdong, Guangxi, Guizhou, Hainan, Hunan, Sichuan, Yunnan); - India, Nepal, Myanmar, Thailand, Vietnam; **Palaearctic.** China (Tibet) [1,35,69,99].
**Host plants. Asteraceae:** *Artemisia* sp. [35]; **Cucurbitaceae:** *Gynostemma pentaphyllum* [16,109]; **Solanaceae:** *Solanum nigrum*, *S. tuberosum*, *S. melongena*, *S. torvum* [16,109], *S. verbascifolium* [16].

54) ***Diekeana maxima* (Weise, 1898)**

*Solanophila maxima* Weise, 1898e [164]: 236. Type locality: India (Assam).

*Epilachna maxima* (Weise [164]): — Li & Cook [21]: 60; Pang & Mao [53]: 134; Pang [58]: 108; Jadwiszczak & Węgrzynowicz [1]: 89; Yu & Pang [118]: 15; Poorani [93]: 43; Kovář [94]: 628; Ren et al. [9]: 288; Pang et al. [69]: 35; Chandra et al. [159]: 417.

*Afissa maxima* (Weise [164]): — Liu [116]: 29.

*Diekeana maxima* (Weise [164]): — Szawaryn et al. [34]: 562; Tomaszewska & Szawaryn [2]: 74; Nakano [16]: 152.

**Distribution. Oriental.** China (Guangdong, Yunnan, Taiwan); - India, Vietnam [1,58,69].
**Host plants. Cucurbitaceae:** *Trichosanthes* sp.; **Solanaceae:** *Solanum verbascifolium* [16].

55) ***Diekeana parainsignis* (Pang & Mao, 1979)**

*Epilachna parainsignis* Pang & Mao, 1979 [53]: 133. Type locality: China (Yunnan). — Wang & Cao [122]: 117; Pang & Zeng [67]: 281; Jadwiszczak & Węgrzynowicz [1]: 102; Kovář [94]: 628; Ren et al. [9]: 292; Zhang & Ou [109]: 56; Pang et al. [69]: 36; Nakano [16]: 155.

*Diekeana parainsignis* (Pang & Mao [53]): — Szawaryn et al. [34]: 563; Tomaszewska & Szawaryn [2]: 74.

**Distribution. Oriental.** China (Guangxi, Guizhou, Yunnan) [69].
**Host plants. Cucurbitaceae:** *Melothria india*; **Solanaceae:** *Solanum torvum* [16,109], *S. verbascifolium* [16]; **Urticaceae:** *Urtica fissa* [16,109].
**Genus *Epilachna* Chevrolat, 1837**

*Epilachna* Chevrolat *in* Dejean, 1837 [156]: 460. Type species: *Coccinella borealis* Fabricius, 1775 [165]: 82. Subsequent designation of Hope [166]: 157. — *Epilachna* sensu stricto Szawaryn et al. [34]: 561; Casey [167]: 103; Li & Cook [21]: 51; Gordon [13]: 37; Pang & Mao [53]: 130; Fürsch [80]: 221; Li [121]: 222; Chakraborty & Biswas [117]: 145; Poorani [93]: 40; Kovář [94]: 626; Ślipiński [31]: 535; Bousquet & Bouchard [158]: 140; Bustamante-Navarrete et al. [168]: 4; Bustamante-Navarrete et al. [169]: 101; Cho [145]: 311; Chandra et al. [159]: 417; González [97]: 535; Bajracharya & Budha [99]: 850; González et al. [98]: 71; Duarte-de-Mélo et al. [17]: 469.

**Remarks.** The genus *Epilachna* currently includes 59 species in China, of which 45 are endemic. Among these endemic species, 28 species are restricted to a single region: Yunnan (8), Taiwan (7), Sichuan (6), Guizhou (2), Tibet (2), and 1 species each in Hainan, Henan, Hubei, and Guangxi. The remaining 17 endemic species range across two to six regions. Overall, the provisional distribution records of *Epilachna* in China are as follows: Sichuan (21), Taiwan (14), Guizhou (13), Guangxi (10), Hubei (7), Tibet (7), Guangdong (6), Hainan (4), Hunan (3), Jiangsu (3), Fujian (2), Shaanxi (2), Shandong (2), and a single species each from Gansu, Henan, and Zhejiang. Host plant records are available for 13 species, revealing associations with 17 plant species from eight families. Urticaceae is the most frequently recorded host family (7 species), followed by Rubiaceae (4) and Asteraceae (3), while Hydrangeaceae, Lamiaceae, and Vitaceae are each represented by a single species. No confirmed host plant records exist for Cucurbitaceae and Poaceae.

**Note.** A total of 14 species, including 11 originally described in *Afissa* and 3 later transferred to *Afissa* in subsequent records, are currently placed in *Epilachna* (sensu Jadwiszczak & Węgrzynowicz [1]). The taxonomic placement of these species should be carefully reassessed in future studies to ensure accurate classification.

56) ***Epilachna adscita* (Mader, 1930)**

*Solanophila adscita* Mader, 1930 [45]: 184. Type locality: China (Sichuan). — Dieke [114]: 118.

*Epilachna adscita* (Mader [45]): — Jadwiszczak & Węgrzynowicz [1]: 32; Pang et al. [69]: 34; Kovář [94]: 626; Nakano [16]: 139.

**Distribution. Oriental.** China (Sichuan) [69].

57) ***Epilachna anthodea* Zeng & Yang, 1996**

*Epilachna anthodea* Zeng & Yang, 1996b [60]: 193, 199. Type locality: China (Guangxi). —Jadwiszczak & Węgrzynowicz [1]: 46; Kovář [94]: 626; Ren et al. [9]: 264; Pang et al. [69]: 34; Nakano [16]: 140.

**Distribution. Oriental**. China (Guangxi, Guizhou) [16,60].

58) ***Epilachna apicilaris* Yu, 2000**

*Epilachna apicilaris* Yu, 2000 [63]: 67. Type locality: China (Henan). — Kovář [94]: 626; Ren et al. [9]: 264; Pang et al. [69]: 34; Nakano [16]: 140.

**Distribution. Oriental**. China (Henan) [69].

59) ***Epilachna arytaenoidea* Zeng, 2000**

*Epilachna arytaenoidea* Zeng, 2000a [64]: 33. Type locality: China (Sichuan). — Pang et al. [69]: 34.

**Distribution. Oriental.** China (Sichuan) [64].

60) ***Epilachna bengalica* (Dieke, 1947)**

*Afissa bengalica* Dieke, 1947 [114]: 130. Type locality: India. — Kapur [49]: 4, 9; Canepari [132]: 58.

*Epilachna bengalica* (Dieke [114]): — Pang & Mao [52]: 324; Li [121]: 400; Canepari [135]: 30; Chakraborty & Biswas [117]: 145; Jadwiszczak & Węgrzynowicz [1]: 39; Poorani [93]: 40; Kovář [94]: 626; Pang et al. [69]: 34; Dorji et al. [26]: 514; Nakano [16]: 140; Bajracharya & Budha [99]: 850.

**Distribution. Oriental.** India, Bhutan, Nepal, Myanmar; **Palaearctic.** China (Tibet) [1,26,69].

61) ***Epilachna bicrescens* (Dieke, 1947)**

*Afissa bicrescens* Dieke, 1947 [114]: 142. Type locality: China (Sichuan).

*Epilachna bicrescens* (Dieke [114]): — Pang & Mao [53]: 152; Xiao & Li [57]: 385; Jadwiszczak & Węgrzynowicz [1]: 40; Kovář [94]: 626; Ren et al. [9]: 266; Pang et al. [69]: 5, 34; Nakano [16]: 140.

**Distribution. Oriental.** China (Hubei, Guangdong, Sichuan, Guizhou) [69].

62) ***Epilachna brachyloba* Zeng & Yang, 1996**

*Epilachna brachyloba* Zeng & Yang, 1996b [60]: 194, 199. Type locality: China (Guangxi). —Jadwiszczak & Węgrzynowicz [1]: 46; Kovář [94]: 626; Pang et al. [69]: 34; Nakano [16]: 141.

*Epilachna brachyfoliata* Zeng & Yang [60]: — an incorrect spelling used by Ren et al. [9]: 266; Zhang & Ou [109]: 56; Pang et al. [69]: 6.

**Distribution. Oriental.** China (Guangxi, Guangdong, Sichuan) [60,69].
**Host plants. Urticaceae:** *Boehmeria* sp. [16,109], *Urtica fissa* [16,60,69], *U. lotabifolia* [16,109].

63) ***Epilachna brivioi* (Bielawski & Fürsch, 1960)**

*Afissa brivioi* Bielawski & Fürsch, 1960 [170]: 70. Type locality: Myanmar.

*Epilachna brivioi* (Bielawski & Fürsch [170]): — Pang & Mao [53]: 148; Wang & Cao [91]: 120; Jadwiszczak & Węgrzynowicz [1]: 46; Poorani [93]: 40; Kovář [94]: 627; Pang et al. [69]: 34; Nakano [16]: 141.

**Distribution. Oriental.** China (Yunnan); - Myanmar [1,69].

64) ***Epilachna chayuensis* Pang & Mao, 1977**

*Epilachna chayuensis* Pang & Mao, 1977 [52]: 324, 327. Type locality: China (Xizang). — Li [121]: 399; Ren et al. [9]: 266; Kovář [94]: 627; Pang et al. [69]: 34; Nakano [16]: 142

**Distribution. Palaearctic.** China (Tibet) [69].

65) ***Epilachna completa* (Dieke, 1947)**

*Afissa completa* Dieke, 1947 [114]: 155. Type locality: China (Sichuan).

*Epilachna completa* (Dieke [114]): — Xiao & Li [57]: 385; Jadwiszczak & Węgrzynowicz [1]: 51; Kovář [94]: 627; Pang et al. [69]: 34; Nakano [16]: 143.

**Distribution. Oriental**. China (Sichuan) [69].

66) ***Epilachna confusa* Li, 1961**

*Epilachna confusa* Li *in* Li & Cook, 1961 [21]: 72. Type locality: China (Taiwan). — Pang & Mao [53]: 158; Pang [58]: 106; Yu & Pang [118]: 14; Jadwiszczak & Węgrzynowicz [1]: 52; Kovář [94]: 627; Ren et al. [9]: 268; Pang et al. [69]: 6, 34; Nakano [16]: 144.

**Distribution. Oriental.** China (Guangdong, Hainan, Guizhou, Taiwan) [21,58,69].

67) ***Epilachna crassimala* Li & Cook, 1961**

*Epilachna crassimala* Li & Cook, 1961 [21]: 68. Type locality: China (Taiwan). — Yu & Pang [118]: 14; Jadwiszczak & Węgrzynowicz [1]: 54; Kovář [94]: 627; Nakano [16]: 144.

*Epilachna cressimala*: An incorrect spelling used by Pang & Mao [53]: 156; Ren et al. [9]: 270; Pang et al. [69]: 34.

**Distribution. Oriental**. China (Taiwan) [69].
**Host plant. Poaceae** [16].

68) ***Epilachna decemguttata* (Weise, 1923)**

*Solanophila decemguttata* Weise, 1923b [44]: 183. Type locality: China (Taiwan).

*Epilachna decemguttata* (Weise [44]): — Li & Cook [21]: 56; Yu & Pang [118]: 15; Jadwiszczak & Węgrzynowicz [1]: 56; Kovář [94]: 627; Pang et al. [69]: 34; Nakano [16].

**Distribution. Oriental.** China (Taiwan) [21,69].

69) ***Epilachna decemmaculata* Redtenbacher, 1844**

*Epilachna decemmaculata* Redtenbacher *in* Kollar & Redtenbacher, 1844 [171]: 564. Type locality: India (Kashmir). — Jadwiszczak & Węgrzynowicz [1]: 56; Poorani [93]: 41; Kovář [94]: 627; Pang et al. [69]: 34; Dorji et al. [26]: 514; Nakano [16]: 144; Bajracharya & Budha [99]: 850.

**Distribution. Oriental.** China (Taiwan); - India, Myanmar, Nepal, Sri Lanka; **Palaearctic.** China (Tibet) [1,26,69].

70) ***Epilachna dictyodroma* Zeng, 2000**

*Epilachna dictyodroma* Zeng, 2000a [64]: 32. Type locality: China (Sichuan). — Ren et al. [9]: 272; Pang et al. [69]: 6; Nakano [16]: 145.

**Distribution. Oriental.** China (Guizhou, Sichuan) [64,69].

71) ***Epilachna dobzhanskyi* (Dieke, 1947)**

*Afissa dobzhanskyi* Dieke, 1947 [114]: 163. Type locality: China (Shandong).

*Epilachna dobzhanskyi* (Dieke [114]): — Xiao & Li [57]: 385; Jadwiszczak & Węgrzynowicz [1]: 59; Kovář [94]: 627; Pang et al. [69]: 34; Nakano [16]: 145.

**Distribution. Oriental.** China (Hubei, Jiangsu, Sichuan, Shandong); **Palaearctic.** China (Shaanxi) [69].

72) ***Epilachna donghoiensis* Hoàng, 1978**

*Epilachna donghoiensis* Hoàng, 1978 [88]: 832. Type locality: Vietnam. — Peng et al. [66]: 89; Jadwiszczak & Węgrzynowicz [1]: 59; Ren et al. [9]: 272; Pang et al. [69]: 34; Nakano [16]: 145.

**Distribution. Oriental.** China (Hainan); - Vietnam [1,66,69,88].

73) ***Epilachna echinata* Pang & Ślipiński, 2012**

*Epilachna echinata* Pang & Ślipiński *in* Pang et al. [69]: 31. Type locality: China (Sichuan). —Nakano [16]: 145.

**Distribution. Oriental.** China (Sichuan); **Palaearctic.** China (Shaanxi) [69].

74) ***Epilachna emeiensis* Zeng, 2000**

*Epilachna emeiensis* Zeng, 2000a [64]: 32. Type locality: China (Sichuan). — Ren et al. [9]: 274; Pang et al. [69]: 6; Nakano [16]: 145.

**Distribution. Oriental.** China (Hubei, Guangxi, Yunnan, Sichuan, Guizhou) [64,69].

75) ***Epilachna erythrotricha* Hoàng, 1978**

*Epilachna erythrotricha* Hoàng, 1978 [88]: 833. Type locality: Vietnam. — Zeng & Pang [143]: 114; Jadwiszczak & Węgrzynowicz [1]: 62; Kovář [94]: 627; Ren et al. [9]: 274; Pang et al. [69]: 6, 35; Nakano [16]: 145.

**Distribution. Oriental.** China (Guangxi, Guizhou, Sichuan, Yunnan); - Vietnam [69,88].
**Host plant. Vitaceae:** *Cayratia japonica* [16].

76) ***Epilachna fenchinica* Pang, 1993**

*Epilachna fenchinica* Pang, 1993 [58]: 109. Type locality: China (Taiwan). — Yu & Pang [118]: 15; Jadwiszczak & Węgrzynowicz [1]: 64; Kovář [94]: 627; Pang et al. [69]: 35; Nakano [16]: 146.

**Distribution. Oriental**. China (Taiwan) [69].

77) ***Epilachna filamentacea* Zeng & Pang, 2002**

*Epilachna filamentacea* Zeng & Pang *in* Pang & Zeng, 2002 [67]: 280. Type locality: China (Guizhou: Maolan Landscape).

**Distribution. Oriental**. China (Guizhou) [67].

78) ***Epilachna formosana* (Weise, 1923)**

*Solanophila formosana* Weise, 1923b [44]: 183. Type locality: China (Taiwan).

*Afissa formosana* (Weise [44]): — Bielawski [120]: 384.

*Epilachna formosana* (Weise [44]): — Li & Cook [21]; 73; Pang [58]: 107; Jadwiszczak & Węgrzynowicz [1]: 66; Kovář [94]: 626; Pang et al. [69]: 35; Nakano [16]: 146.

**Distribution. Oriental.** China (Taiwan) [58,69].

79) ***Epilachna freyana* Bielawski, 1965**

*Epilachna freyana* Bielawski, 1965c [50]: 219. Type locality: China (Sichuan). — Pang & Mao [53]: 153; Wang & Cao [91]: 120; Jadwiszczak & Węgrzynowicz [1]: 67; Ren et al. [9]: 276; Pang et al. [69]: 6, 35; Nakano [16]: 146.

**Distribution. Oriental.** China (Sichuan, Yunnan) [50,69].

80) ***Epilachna gloiera* Xiao, 1992**

*Epilachna gloiera* Xiao *in* Xiao & Li [57]: 386, 390. Type locality: China (Sichuan). — Jadwiszczak & Węgrzynowicz [1]: 70; Kovář [94]: 626; Pang et al. [69]: 35; Nakano [16]: 148.

*Epilachna globiera* (error typogr.!): — Xiao *in* Xiao & Li [57]: 386.

**Distribution. Oriental.** China (Sichuan) [69].

81) ***Epilachna gokteika* (Kapur, 1961)**

*Afissa gokteika* Kapur, 1961a [172]: 139. Type locality: Myanmar.

*Epilachna gokteika* (Kapur [172]): — Peng et al. [66]: 90; Jadwiszczak & Węgrzynowicz [1]: 71; Poorani [93]: 42; Ren et al. [9]: 278; Pang et al. [69]: 35; Nakano [16]: 148.

**Distribution. Oriental.** China (Guangxi, Hainan); - Myanmar [1,66].

82) ***Epilachna hendecaspilota* (Mader, 1927)**

*Solanophila hendecaspilota* Mader, 1927b [173]: 44. Type locality: Asia (India, China, Myanmar, Java, Borneo).

*Afissa hendecaspilota* (Mader [173]): — Canepari [132]: 59.

*Epilachna hendecaspilota* (Mader [173]): — Pang & Mao [53]: 155; Pang [58]: 108; Yu & Pang [118]: 15; Jadwiszczak & Węgrzynowicz [1]: 74; Poorani [93]: 42; Kovář [94]: 626; Pang et al. [69]: 35; Nakano [16]: 148; Bajracharya & Budha [99]: 851.

**Distribution. Oriental.** China (Taiwan); - India, Myanmar, Sri Lanka, Nepal, Thailand, Vietnam, Philippines, Indonesia (Java, Borneo) [1,58,69,99].

83) ***Epilachna hingstoni* (Kapur, 1963)**

*Afissa hingstoni* Kapur, 1963 [49]: 10. Type locality: China (Tibet). — Canepari [132]: 58.

*Epilachna hingstoni* (Kapur [49]): — Miyatake [89]: 25; Jadwiszczak & Węgrzynowicz [1]: 74; Poorani [93]: 42; Kovář [94]: 626; Ren et al. [9]: 280; Canepari [15]: 365; Pang et al. [69]: 35; Nakano [16]: 148; Bajracharya & Budha [99]: 851.

**Distribution. Oriental.** China (Yunnan); - India, Nepal; **Palaearctic.** China (Tibet) [1,69,99].

84) ***Epilachna jianchuanensis* Cao & Xiao, 1984**

*Epilachna jianchuanensis* Cao & Xiao, 1984 [55]: 113,127. Type locality: China (Yunnan). — Wang & Cao [91]: 120; Jadwiszczak & Węgrzynowicz [1]: 78; Kovář [94]: 627; Ren et al. [9]: 283; Zhang & Ou [109] 53; Pang et al. [69]: 35.

**Distribution. Oriental.** China (Yunnan) [69].
**Host plant. Rubiaceae**: *Paederia scandens* [109].

85) ***Epilachna kunmingensis* Yi, He & Xiao, 2013**

*Epilachna kunmingensis* Yi, He & Xiao *in* Yi et al. 2013 [70]: 114. Type locality: China (Yunnan). — Nakano [16]: 149.

**Distribution. Oriental.** China (Yunnan) [70].
**Host plant. Rubiaceae**: *Rubia oncotricha* [70].

86) ***Epilachna lenta* (Weise, 1902)**

*Solanophila lenta* Weise, 1902b [174]: 495. Type locality: Vietnam.

*Epilachna lenta* (Weise [174]): — Bielawski [50]: 215; Jadwiszczak & Węgrzynowicz [1]: 83; Ren et al. [9]: 284; Nakano [16]: 150.

**Distribution. Oriental.** China (Guangdong); - Vietnam; **Palaearctic.** China (Gansu) [1,9].

87) ***Epilachna lichuaniensis* Xiao, 1992**

*Epilachna lichuaniensis* Xiao *in* Xiao & Li [57]: 387, 390. Type locality: China (Hubei). — Jadwiszczak & Węgrzynowicz [1]: 84; Kovář [94]: 628; Pang et al. [69]: 13, 35; Nakano [16]: 150.

**Distribution. Oriental.** China (Hubei, Sichuan, Yunnan) [69].

88) ***Epilachna longissima* (Dieke, 1947)**

*Afissa longissima* Dieke, 1947 [114]: 148. Type locality: China (Taiwan).

*Epilachna longissima* (Dieke [114]): — Li & Cook [21]; 67; Pang & Mao [53]: 157; Pang [58]: 106; Yu & Pang [118]: 15; Jadwiszczak & Węgrzynowicz [1]: 84; Kovář [94]: 628; Ren et al. [9]: 284; Pang et al. [69]: 35; Nakano [16]: 150.

**Distribution. Oriental.** China (Fujian, Taiwan, Sichuan, Yunnan) [58,69].
**Host plant. Lamiaceae:** *Callicarpa formosana* [16].

89) ***Epilachna lugubris* (Dieke, 1947)**

*Afissa lugubris* Dieke, 1947 [114]: 157. Type locality: China (Shandong).

*Epilachna lugubris* (Dieke [114]): — Jadwiszczak & Węgrzynowicz [1]: 85; Kovář [94]: 628; Pang et al. [69]: 35; Nakano [16]: 150.

**Distribution. Oriental.** China (Jiangsu); **Palaearctic**. China (Shandong) [1,69].

90) ***Epilachna maculicollis* (Sicard, 1922)**

*Solanophila nilghirica* v. *maculicollis* Sicard, 1922 [175]: 131. Type locality: China (Taiwan).

*Afissa maculicollis* (Sicard [175]): — Dieke [114]: 131.

*Epilachna maculicollis* (Sicard [175]): — Li & Cook [21]: 61; Pang & Mao [53]: 140; Pang [58]: 108; Yu & Pang [118]: 15; Zheng & Pang [143]: 118; Pang & Zeng [67]: 281; Jadwiszczak & Węgrzynowicz [1]: 87; Kovář [94]: 628; Ren et al. [9]: 286; Pang et al. [69]: 13, 35; Nakano [16]: 150.

**Distribution. Oriental.** China (Zhejiang, Guangdong, Guangxi, Taiwan, Yunnan, Guizhou) [58,69].
**Host plants. Urticaceae:** *Boehmeria* sp., *B. nivea*, *Debregeasia orientalis*, *Elatostema platyphyllum* [16].

91) ***Epilachna maculivestis* Mulsant, 1853**

*Epilachna maculivestis* Mulsant, 1853 [37]: 251. Type locality: China (Tibet). — Jadwiszczak & Węgrzynowicz [1]: 87; Poorani [93]: 43; Kovář [94]: 628; Pang et al. [69]: 35; Dorji et al. [26]: 515; Nakano [16]: 151; Bajracharya & Budha [99]: 851.

**Distribution. Oriental**. Bhutan, India, Nepal; **Palaearctic**. China (Tibet) [1,26,69,99].

92) ***Epilachna maolanensis* Zeng & Pang, 2002**

*Epilachna maolanensis* Zeng & Pang *in* Pang & Zeng, 2002 [67]: 281. Type locality: China (Guizhou: Maolan Landscape).

**Distribution. Oriental**. China (Guizhou) [67].

93) ***Epilachna marginicollis* (Hope, 1831)**

*Coccinella marginicollis* Hope, 1831 [155]: 31. Type locality: Nepal.

*Afissa marginicollis* (Hope [155]): — Kapur [82]: 315; Canepari [132]: 61.

*Epilachna marginicollis* (Hope [155]): — Mulsant [128]: 728; Pang & Mao [52]: 324; Li [121]: 399; Canepari [135]: 29; Chakraborty & Biswas [117]: 146; Jadwiszczak & Węgrzynowicz [1]: 88; Poorani [93]: 43; Kovář [94]: 628; Ren et al. [9]: 288; Canepari [15]: 365; Pang et al. [69]: 35; Dorji et al. [26]: 515; Nakano [16]: 151; Bajracharya & Budha [99]: 851.

**Distribution. Oriental.** Bhutan, India, Myanmar, Nepal; **Palaearctic.** China (Tibet) [1,69,99].
**Host plant. Cucurbitaceae** [16].

94) ***Epilachna menglunensis* Hu & Zhang, 1997**

*Epilachna menglunensis* Hu & Zhang, 1997 [61]: 30, 33. Type locality: China (Yunnan). — Pang et al. [69]: 35; Nakano [16]: 153.

**Distribution. Oriental.** China (Yunnan) [61,69].

95) ***Epilachna microgenitalia* Li, 1961**

*Epilachna microgenitalia* Li *in* Li & Cook, 1961 [21]: 70. Type locality: China (Taiwan). — Xiao & Li [57]: 385; Yu & Pang [118]: 15; Jadwiszczak & Węgrzynowicz [1]: 91; Kovář [94]: 628; Pang et al. [69]: 13, 35; Nakano [16]: 153.

**Distribution. Oriental.** China (Guangxi, Sichuan, Taiwan) [21,69].

96) ***Epilachna mirabiloides* (Dieke, 1947)**

*Afissa mirabiloides* Dieke, 1947 [114]: 131. Type locality: China (Sichuan).

*Epilachna mirabiloides* (Dieke [114]): — Jadwiszczak & Węgrzynowicz [1]: 91; Kovář [94]: 628; Pang et al. [69]: 35; Nakano [16]: 153.

**Distribution. Oriental.** China (Sichuan); - Vietnam [1,69].

97) ***Epilachna monandrum* Yi, He & Xiao, 2013**

*Epilachna monandrum* Yi, He & Xiao *in* Yi et al. 2013 [70]: 112: Type locality: China (Yunnan). — Nakano [16]: 153.

**Distribution. Oriental.** China (Yunnan) [70].
**Host plant. Urticaceae:** *Elatostema monandrum* [70].

98) ***Epilachna mushana* Li, 1961**

*Epilachna mushana* Li *in* Li & Cook, 1961 [21]: 71. Type locality: China (Taiwan). — Yu & Pang [118]: 15; Jadwiszczak & Węgrzynowicz [1]: 93; Kovář [94]: 628; Pang et al. [69]: 35; Nakano [16]: 154.

**Distribution. Oriental.** China (Taiwan) [69].

99) ***Epilachna ocellataemaculata* (Mader, 1930)**

*Solanophila ocellatae-maculata* Mader, 1930 [45]: 183. Type locality: China (Yunnan).

*Epilachna ocellataemaculata* (Mader [45]): — Pang & Mao [53]: 150; Xiao & Li [57]: 385; Wang & Cao [91]:120; Wang & Cao [122]: 118; Jadwiszczak & Węgrzynowicz [1]: 98; Kovář [94]: 628; Ren et al. [9]: 290; Zhang & Ou [109]: 53; Pang et al. [69]: 20, 35; Katoh et al. [22]: 822; Nakano [16]: 154.

**Distribution. Oriental.** China (Guizhou, Hubei, Hunan, Yunnan, Sichuan); - Vietnam [1,69].
**Host plants. Asteraceae** [22], *Artemisia dubia*, *A. lavandulaefolia* [16,109], *A. rubripes* [16].

100) ***Epilachna ocreata* Zeng & Yang, 1996**

*Epilachna ocreata* Zeng & Yang, 1996b [60]: 196, 200. Type locality: China (Guangxi). — Kovář [94]: 628; Ren et al. [9]: 292; Pang et al. [69]: 35; Nakano [16]: 154.

**Distribution. Oriental.** China (Guangxi, Guizhou, Hunan) [60,69].
**Host plant. Urticaceae** [16].

101) ***Epilachna paling* Yu, 2001**

*Epilachna paling* Yu, 2001 [176]: 99. Type locality: China (Taiwan). — Jadwiszczak & Węgrzynowicz [1]: 101; Kovář [94]: 628; Pang et al. [69]: 35; Nakano [16]: 154.

**Distribution. Oriental.** China (Taiwan) [69].

102) ***Epilachna paraglobiera* Peng, Pang & Pang, 2001**

*Epilachna paraglobiera* Peng, Pang & Pang *in* Peng et al. 2001 [66]: 88, 91. Type locality: China (Hainan). — Nakano [16]: 155.

**Distribution. Oriental.** China (Hainan) [66,69].

103) ***Epilachna pingbianensis* Pang & Mao, 1979**

*Epilachna pingbianensis* Pang & Mao, 1979 [53]: 146. Type locality: China (Yunnan). — Jadwiszczak & Węgrzynowicz [1]: 106; Kovář [94]: 628; Ren et al. [9]: 294; Pang et al. [69]: 36; Nakano [16]: 155.

**Distribution. Oriental.** China (Yunnan) [69].

104) ***Epilachna provisoria* (Dieke, 1947)**

*Afissa provisoria* Dieke, 1947 [114]: 151. Type locality: China (Sichuan).

*Epilachna provisoria* (Dieke [114]): — Jadwiszczak & Węgrzynowicz [1]: 108; Kovář [94]: 628; Pang et al. [69]: 36; Nakano [16]: 156.

**Distribution. Oriental.** China (Sichuan) [69].

105) ***Epilachna pui* Hu & Zhang, 1997**

*Epilachna pui* Hu & Zhang, 1997 [61]: 31, 33. Type locality: China (Yunnan). — Pang et al. [69]: 36; Nakano [16]: 156.

**Distribution. Oriental**. China (Yunnan) [61,69].

106) ***Epilachna rubiacis* Cao & Xiao, 1984**

*Epilachna rubiacis* Cao & Xiao, 1984 [55]: 112,126. Type locality: China (Yunnan). — Wang & Cao [122]: 117; Wang & Cao [91]: 120; Jadwiszczak & Węgrzynowicz [1]: 111; Kovář [94]: 628; Zhang & Ou [109]: 53; Pang et al. [69]: 36; Nakano [16]: 157.

**Distribution. Oriental**. China (Yunnan) [69].
**Host plants. Rubiaceae**: *Galium aparine*, *Rubia cordifolia* [16,109].

107) ***Epilachna sauteri* (Weise, 1923)**

*Solanophila sauteri* Weise, 1923b [44]: 182. Type locality: China (Taiwan).

*Epilachna sauteri* (Weise [44]): — Li & Cook [21]; 75; Pang & Mao [53]: 147; Yu & Pang [118]: 15; Jadwiszczak & Węgrzynowicz [1]: 113; Kovář [94]: 628; Ren et al. [9]: 296; Pang et al. [69]: 22, 36; Nakano [16]: 157.

**Distribution. Oriental.** China (Fujian, Guizhou, Hunan, Hubei, Jiangsu, Sichuan, Taiwan); **Palaearctic.** Japan [1,66,69].

**Host plant. Hydrangeaceae**: *Hydrangea chinensis* [16].

108) ***Epilachna shennongjiaensis* Peng, Pang & Ren, 2000**

*Epilachna shennongjiaensis* Peng, Pang & Ren *in* Peng et al. 2000 [177]: 133, 135. Type locality: China (Hubei: Shennongjia Mt.).

**Distribution. Oriental.** China (Hubei) [177].

109) ***Epilachna sichuana* Pang & Ślipiński, 2012**

*Epilachna sichuana* Pang & Ślipiński *in* Pang et al. 2012 [69]: 27. Type locality: China (Sichuan). —Nakano [16]: 157.

**Distribution. Oriental.** China (Sichuan) [69].

110) ***Epilachna siphonechinulata* Zeng & Yang, 1996**

*Epilachna siphonechinulata* Zeng & Yang, 1996b [60]: 197, 200. Type locality: China (Guangxi). —Pang & Zeng [67]: 282; Jadwiszczak & Węgrzynowicz [1]: 117; Ren et al. [9]: 298; Pang et al. [69]: 36; Nakano [16]: 157.

**Distribution. Oriental.** China (Guangxi) [60,69].

111) ***Epilachna sociolamina* Li, 1961**

*Epilachna sociolamina* Li *in* Li & Cook, 1961 [21]: 64. Type locality: China (Taiwan). — Yu & Pang [118]: 15; Jadwiszczak & Węgrzynowicz [1]: 117; Kovář [94]: 628; Pang et al. [69]: 36; Nakano [16]: 158.

**Distribution. Oriental**. China (Taiwan) [69].

112) ***Epilachna spiraloides* Cao & Xiao, 1984**

*Epilachna spiraloides* Cao & Xiao, 1984 [55]: 114, 127. Type locality: China (Yunnan). — Jadwiszczak & Węgrzynowicz [1]: 118; Kovář [94]: 628; Nakano [16]: 158.

*Epilachna spiroloides* Cao & Xiao [55]: — an incorrect spelling used by Pang et al. [69]: 36.

**Distribution. Oriental**. China (Yunnan) [69].

113) ***Epilachna tianpingiensis* Pang & Mao, 1979**

*Epilachna tianpingiensis* Pang & Mao, 1979 [53]: 142. Type locality: China (Guangxi). — Zhang & Qi [142]: 250; Zheng & Pang [143]: 118; Pang & Zeng [67]: 282; Jadwiszczak & Węgrzynowicz [1]: 122; Kovář [94]: 628; Ren et al. [9]: 300; Pang et al. [69]: 22, 36; Nakano [16]: 158.

**Distribution. Oriental.** China (Guangdong, Guangxi, Guizhou) [53,69].

114) ***Epilachna tridecimmaculosa* Pang & Mao, 1977**

*Epilachna tridecimmaculosa* Pang & Mao, 1977 [52]: 326, 328. Type locality: China (Xizang). — Li [121]: 398; Ren et al. [9]: 300; Jadwiszczak & Węgrzynowicz [1]: 124; Kovář [94]: 628; Nakano [16]: 158.

*Epilachna tredecimmaculosa* Pang & Mao [52]: — an incorrect spelling used by Pang & Mao [52]: 324.

*Epilachna tridecimaculata* Pang & Mao [52]: — an incorrect spelling used by Pang et al. [69]: 36.

**Distribution. Palaearctic**. China (Tibet) [69].
**Genus *Epiverta* Dieke, 1947**

*Epiverta* Dieke, 1947 [114]: 169. Type species: *Solanophila chelonia* Mader, 1933 [46]: 79. Original designation. — Pang & Mao [53]: 159; Jadwiszczak & Węgrzynowicz [1]: 208; Kovář [94]: 631; Katoh et al. [22]: 827; Szawaryn et al. [34]: 556, 565; Tomaszewska & Szawaryn [2]: 47; Tomaszewska et al. [72]: 2; Nakano [16]: 167.

**Remarks.** The genus *Epiverta* includes four described species endemic to China and predominantly distributed in Oriental region, with the exception of *Epiverta supinata*, which extends into the Palaearctic region (Tibet). Host plant data is available only for *E. chelonia*, which feeds on Asteraceae and Ranunculaceae.

115) ***Epiverta albopilosa* Tomaszewska, Huo, Szawaryn & Wang, 2017**

*Epiverta albopilosa* Tomaszewska, Huo, Szawaryn & Wang *in* Tomaszewska et al. 2017 [72]: 6. Type locality: China (Yunnan). — Nakano [16]: 168.

**Distribution. Oriental.** China (Yunnan) [72].

116) ***Epiverta angusta* Tomaszewska, Huo, Szawaryn & Wang, 2017**

*Epiverta angusta* Tomaszewska, Huo, Szawaryn & Wang *in* Tomaszewska et al. 2017 [72]: 7. Type locality: China (Sichuan). — Nakano [16]: 168.

**Distribution. Oriental.** China (Sichuan) [72].

117) ***Epiverta supinata* Tomaszewska, Huo, Szawaryn & Wang, 2017**

*Epiverta supinata* Tomaszewska, Huo, Szawaryn & Wang *in* Tomaszewska et al. 2017 [72]: 9. Type locality: China (Sichuan). — Nakano [16]: 168.

**Distribution. Oriental.** China (Sichuan, Yunnan); **Palaearctic.** China (Tibet) [72].

118) ***Epiverta chelonia* (Mader, 1933)**

*Solanophila chelonia* Mader, 1933 [46]: 79. Type locality: China (Yunnan).

*Epiverta chelonia* (Mader [46]): — Bielawski, 1960 [178]: 441; Pang & Mao [53]: 159; Jadwiszczak & Węgrzynowicz [1]: 208; Katoh et al. [22]: 827; Szawaryn et al. [34]: 549; Tomaszewska & Szawaryn [2]: 49; Tomaszewska et al. [72]: 3; Nakano [16]: 167; Zhang et al. 2023 [179]: 7.

**Distribution. Oriental.** China (Sichuan, Yunnan) [1,72].
**Host plants. Asteraceae** [22], *Artemisia* sp. [16,53]; **Ranunculaceae** [22], *Anemone tomentosa*, *Eriocapitella rivularis* [16,53].
**Genus *Henosepilachna* Li, 1961**

*Henosepilachna* Li *in* Li & Cook, 1961 [21]: 35. Type species: *Coccinella sparsa* Herbst, 1786 [180] (= *Coccinella vigintioctopunctata* Fabricius, 1775 [165]). Original designation. — Pang & Mao [53]: 107; Fürsch [80]: 239; Li [149]: 210; Yu & Pang [118]: 13; Thapa [124]: 146; Chakraborty & Biswas [117]: 147; Jadwiszczak & Wegrzynowicz [1]: 132; Poorani [93]: 45; Kovář [94]: 629; Nattier et al. 2015 [181]: 314; Szawaryn et al. [34]: 560; Tomaszewska & Szawaryn [2]: 56; Ahmed et al. [112]: 1754; Cho [145]: 311; Nakano [16]: 159; Das et al. [35]: 41; Chandra et al. [159]: 417; Bajracharya & Budha [99]: 852; Iqbal et al. [20]: 80; Duarte-de-Mélo et al. [17]: 470; Ye et al. [75]: 333.

*Subafissa* Bielawski, 1963 [182]: 437. Type species: *Epilachna papuensis* Crotch, 1874 [38]: 79. — Jadwiszczak & Węgrzynowicz [1]: 183. Synonymized by Szawaryn et al. [34]: 565.

**Remarks.** The genus *Henosepilachna* currently includes 233 species, predominantly found in the Afrotropical region (122 species), followed by the Oriental (80), Australasia (26), Palaearctic (13), and Neotropical (1) regions. The Chinese fauna comprises 29 species, of which 11 are endemic. Provisional regional distribution records by region are as follows: Yunnan (17), Guangxi (11), Taiwan (10), Guizhou (10), Tibet (9), Hainan (5), Guangdong (5), Beijing (3), Sichuan (3), Zhejiang (3), Hebei (2), Henan (2), Hubei (2), Shandong (2), Shanxi (2), Shaanxi (2), Fujian (2), Jiangsu (2), Liaoning (2), Anhui (1), Chongqing (1), Gansu (1), Hong Kong (1), Hunan (1), Jilin (1), Jiangxi (1), and Heilongjiang (1).

Host plants records are available for 15 species, revealing associations with 112 plant species from 19 families. Solanaceae is the most frequently recorded host family (43 species), followed by Cucurbitaceae (29), Fabaceae (10), Asteraceae (9), Brassicaceae (6) and Poaceae (3). The families Adoxaceae, Apocynaceae, Araliaceae, Boraginaceae, Convolvulaceae, Dennstaedtiaceae, Elaeagnaceae, Juncaceae, Lythraceae, Portulacaceae, Polygonaceae, Schisandraceae, and Urticaceae are each represented by a single species.

119) ***Henosepilachna aduncfolia* Zeng, 2000**

*Henosepilachna aduncfolia* Zeng, 2000c [65]: 136. Type locality: China (Zhejiang).

**Distribution. Oriental.** China (Zhejiang) [65].

120) ***Henosepilachna boisduvali* (Mulsant, 1850)**

*Epilachna boisduvali* Mulsant, 1850 [128]: 765. Type locality: Australia. — Katakura et al. [23]: 330; Ślipiński [31]: 541.

*Epilachna montrouzieri* Fauvel, 1862 [183]: 57 Type locality: New Caledonia. Synonymized by Korschefsky [76]: 32.

*Epilachna montrouzieri* var. *fijiensis* Crotch, 1874 [38]: 89. Type locality: Fiji Is.

*Epilachna boisduvali chabuana* Dieke, 1947 [114]: 80. Type locality: India (Assam).

*Epilachna boisduvali samoana* Dieke, 1947 [114]: 81. Type locality: Samoa Islands, Tutuila, Vaitogi.

*Epilachna boisduvali tokarana* Nakane & Araki, 1959 [184]: A45. Type locality: Tokara Islands (Nakanoshima).

*Henosepilachna boisduvali* (Mulsant [128]): — Li & Cook [21]: 47; Li [149]: 219; Yu & Pang [118]: 13; Kobayashi et al. [161]: 148; Chakraborty & Biswas [117]: 148; Jadwiszczak & Węgrzynowicz [1]: 139; Poorani [93]: 46; Kovář [94]: 629; Ren et al. [9]: 302; Katoh et al. [22]: 822; Nattier et al. [181]: 314; Szawaryn [150]: 161; Patil & Gaikwad, 2023 [185]: 22862; Nakano [16]: 159; Iqbal et al. [20]: 94; Ye et al. [75]: 337.

**Distribution. Australasia.** Australia, New Guinea, New Hebrides, New Caledonia, Samoa Is., Fiji Is.; **Oriental.** China (Taiwan, Hainan, Guizhou); - India, Vietnam, Philippines, Pakistan, Indonesia (West Timor); **Palaearctic.** Japan (Ryukyu Islands) [1,20,75,181].
**Host plants. Apocynaceae:** *Calotropis procera* [20]; **Cucurbitaceae** [22,161], *Diplocyclos palmatus* [16], *Momordica charantia* [20], *Mukia javanica* [23]; **Solanaceae:** *Solanum nigrum* [16].

121) ***Henosepilachna dodecastigma* (Wiedemann, 1823)**

*Coccinella dodecastigma* Wiedemann, 1823 [186]: 73. Type locality: India.

*Epilachna dodecastigma* (Wiedemann [186]): — Kapur, 1963 [49]: 4, 6; Kapur [85]: 150.

*Henosepilachna dodecastigma* (Wiedemann [186]): — Pang & Mao [52]: 324; Li [121]: 400; Canepari [132]: 56; Chakraborty & Biswas [117]: 148; Jadwiszczak & Węgrzynowicz [1]: 144; Poorani [93]: 46; Ren et al. [9]: 302; Nakano [16]: 160; Bajracharya & Budha [99]: 853; Iqbal et al. [20]: 91; Ye et al. [75]: 338.

**Distribution. Oriental.** Bangladesh, Nepal, India, Indonesia, Pakistan, Myanmar; **Palaearctic.** China (Tibet) [1,16,20,75,99].
**Host plants. Cucurbitaceae:** *Cucurbita moschata* [20], *Luffa cylindrica* [16]; **Solanaceae:** *Withania somnifera* [20].

122) ***Henosepilachna genitalis* Hoàng, 1977**

*Henosepilachna genitalis* Hoàng, 1977 [87]: 139. Type locality: Vietnam (Thạnh Hóa, Lang An). — Jadwiszczak & Węgrzynowicz [1]: 149; Ye et al. [75]: 339.

**Distribution. Oriental**. China (Yunnan), Vietnam [1,75,87].

123) ***Henosepilachna hornoformis* Ye, Chen & Wang, 2026**

*Henosepilachna hornoformis* Ye, Chen & Wang *in* Ye et al. 2026 [75]: 340. Type locality: China (Yunnan)

**Distribution. Oriental.** China (Guizhou, Yunnan) [75].

124) ***Henosepilachna indica* (Mulsant, 1850)**

*Epilachna indica* Mulsant, 1850 [128]: 776. Type locality: ‘Indes’. — Kapur [49]: 4, 7; Kapur [83]: 135.

*Epilachna indica* var. *ceylonica* Weise, 1901a [115]: 418. Type locality: Sri Lanka.

*Epilachna tertia* Dieke, 1947 [114]: 66. Type locality: India (Assam). Synonymized by Kapur [83]: 135.

*Henosepilachna indica* (Mulsant [128]): — Pang & Mao [53]: 114; Chakraborty & Biswas [117]: 148; Jadwiszczak & Węgrzynowicz [1]: 154; Poorani [93]: 47; Kovář [94]: 629; Ren et al. [9]: 304; Zhang & Ou [109]: 57; Katoh et al. [22]: 822; Dorji et al. [26]: 516; Nakano [16]: 160; Das et al. [35]: 41; Chandra et al. [159]: 417; Iqbal et al. [20]: 92; Ye et al. [75]: 341.

**Distribution. Oriental.** China (Yunnan, Guizhou, Guangxi, Hainan, Taiwan); - Bhutan, India, Nepal, Laos, Myanmar, Vietnam [1,9,20,75].
**Host plants. Solanaceae [22,26]**, *Solanum torvum* [16,109], *S. verbascifolium* [16].

125) ***Henosepilachna intriogibbera* Yu & Pang, 1993**

*Henosepilachna intriogibbera* Yu & Pang, 1993: 501. Type locality: China. — Kovář [94]: 629; Ren et al. [9]: 304; Nakano [16]: 160; Ye et al. [75]: 342.

**Distribution. Oriental**. China (Guangdong, Guangxi) [9,75].

126) ***Henosepilachna kaszabi* (Bielawski & Fürsch, 1960)**

*Epilachna kaszabi* Bielawski & Fürsch, 1960 [170]: 68. Type locality: Myanmar. — Kapur [85]: 157.

*Henosepilachna kaszabi* (Bielawski & Fürsch [170]): — Jadwiszczak & Węgrzynowicz [1]: 156; Poorani [93]: 47; Kovář [94]: 629; Ren et al. [9]: 304; Katoh et al. [22]: 822; Szawaryn et al. [34]: 560; Tomaszewska & Szawaryn [2]: 56; Halim et al. 2017 [187]: 815; Nakano [16]: 160; Ye et al. [75]: 343.

**Distribution. Oriental.** China (Yunnan, Guizhou, Guangdong, Guangxi, Hainan); - India, Myanmar, Philippines, Thailand, Vietnam [1,16,75].
**Host plants. Cucurbitaceae** [22]; **Solanaceae:** *Solanum verbascifolium* [16].

127) ***Henosepilachna laokayensis* Hoàng, 1977**

*Henosepilachna laokayensis* Hoàng, 1977 [87]: 144. Type locality: Vietnam (Hoàng Len Shon, Lao Kay, 12 lern W from Shapa). — Jadwiszczak & Węgrzynowicz [1]: 157; Tomaszewska & Szawaryn [2]: 56; Ye et al. [75]: 344.

**Distribution. Oriental**. China (Yunnan); –Vietnam [1,2,75].

128) ***Henosepilachna libera* (Dieke, 1947)**

*Epilachna libera* Dieke, 1947 [114]: 85. Type locality: China (Sichuan).

*Henosepilachna libera* (Dieke [114]): — Pang & Mao [53]: 115; Pang [58]: 106; Yu & Pang [118]: 13; Zeng [188]: 42; Jadwiszczak & Węgrzynowicz [1]: 157; Kovář [94]: 629; Ren et al. [9]: 306; Nakano [16]: 161; Ye et al. [75]: 345.

**Distribution. Oriental.** China (Guizhou, Guangxi, Sichuan, Yunnan, Taiwan) [1,58,75].
**Host plant. Cucurbitaceae:** *Thladiantha dubia* [16].

129) ***Henosepilachna maunsonica* Jadwiszczak & Węgrzynowicz, 2003**

*Epilachna freudei* Fürsch, 1959 [77]: 7. Type locality: Vietnam.

*Henosepilachna freudei* (Fürsch [77]): — Zeng [65]: 139.

*Henosepilachna maunsonica* Jadwiszczak & Węgrzynowicz, 2003 [1,9,75]: 158. The replacement name for *Epilachna freudei* Fürsch, 1959 [77]. — Kovář [94]: 629; Ren et al. [9]: 302; Katoh et al. [22]: 822; Nakano [16]: 161; Ye et al. [75]: 346.

**Distribution. Oriental**. China (Guangxi); –Vietnam [1,9,75].
**Host plant. Solanaceae** [22].

130) ***Henosepilachna megista* Ye, Chen & Wang, 2026**

*Henosepilachna megista* Ye, Chen & Wang *in* Ye et al. 2026 [75]: 347. Type locality: China (Xizang).

**Distribution. Palaearctic.** China (Xizang) [75].

131) ***Henosepilachna ocellata* (Redtenbacher, 1844)**

*Epilachna ocellata* Redtenbacher *in* Kollar & Redtenbacher, 1844 [171]: 563. Type locality: India (Kashmir). — Kapur [81]: 18; Kapur [82]: 313.

*Epilachna oculea* Mulsant, 1850 [128]: 791. Type locality: Nepal. Synonymized by Crotch [38].

*Henosepilachna ocellata* (Redtenbacher [171]): — Pang & Mao [52]: 324; Li [121]: 401; Canepari [132]: 56; Chakraborty & Biswas [117]: 148; Jadwiszczak & Węgrzynowicz [1]: 163; Poorani [93]: 47; Kovář [94]: 629; Ren et al. [9]: 306; Hayat & Khan [133]: 348; Tomaszewska & Szawaryn [2]: 56; Nakano [16]: 161; Sajan et al. [25]: 1244; Dorji et al. [26]: 517; Bajracharya & Budha [99]: 853; Iqbal et al. [20]: 90; Ye et al. [75]: 349.

**Distribution. Oriental.** China (Yunnan); - Bhutan, Nepal, India, Pakistan; **Palaearctic.** China (Tibet) [1,16,20,75,99].
**Host plants. Asteraceae:** *Artemisia rubripes* [16]; **Cucurbitaceae:** *Cucumis sativus* [25].

132) ***Henosepilachna operculata* (Liu, 1950)**

*Epilachna operculata* Liu, 1950 [48]: 162. Type locality: China (Beijing).

*Henosepilachna operculata* (Liu [48]): — Pang & Mao [53]: 110; Xiao & Li [57]: 384; Yu [189]: 240; Jadwiszczak & Węgrzynowicz [1]: 163; Kovář [94]: 629; Nakano [16]: 161; Ye et al. [75]: 349.

**Distribution. Palaearctic.** China (Beijing, Fujian) [1,48,75].
**Host plant. Cucurbitaceae:***Actinostemma lobatum* [16].

133) ***Henosepilachna processa* Li & Cook, 1961**

*Epilachna wissmanni* ab. *processa* Weise, 1908 [190]: 217. *Note:* Infrasubspecific name, unavailable in zoological nomenclature, validated by Li & Cook [21]: 46.

*Epilachna processa* Weise: — Kapur [85]: 155.

*Henosepilachna processa* Li & Cook, 1961 [21]: 45. — Pang & Mao [53]: 116; Yu & Pang [118]: 13; Yu [189]: 239; Chakraborty & Biswas [117]: 149; Jadwiszczak & Wegrzynowicz [1]: 164; Kovář [94]: 629; Ren et al. [9]: 306; Canepari [15]: 366; Dorji et al. [26]: 518; Nakano [16]: 161; Poorani et al. [18]: 544; Bajracharya & Budha [99]: 853; Ye et al. [75]: 350.

**Distribution. Oriental.** China (Yunnan, Taiwan); - Bhutan, Nepal, India, Thailand, Myanmar, Vietnam, Philippines, Indonesia [1,16,18,75,99].

134) ***Henosepilachna pusillanima* (Mulsant, 1850)**

*Epilachna pusillanima* Mulsant, 1850 [128]: 784. Type locality: Indonesia (Java). — Booth & Pope [152]: 346; Shirai & Katakura [107]: 76; Katakura et al. [23]: 330, 333.

*Epilachna pusillanima* var. *languens* Mulsant, 1850 [128]: 786. Type locality: India.

*Epilachna dentulata* Dieke, 1947 [114]: 46. Type locality: Vietnam. Synonymized by Li & Cook [21]: 42.

*Epilachna dentulata* subsp. *parvinotata* Dieke, 1947 [114]: 47. Type locality: Philippine Islands. Synonymized By Li & Cook [21]: 42.

*Henosepilachna pusillanima* (Mulsant [128]): — Li & Cook [21]: 42; Pang & Mao [53]: 116; Miyatake [89]: 22; Yu & Pang [118]: 13; Kobayashi et al. [161]: 148; Yu [189]: 241; Zeng [188]: 43; Jadwiszczak & Węgrzynowicz [1]: 165; Poorani [93]: 48; Ren et al. [9]: 308; Katoh et al. [22]: 822; Dorji et al. [26]: 518; Nakano [16]: 162; Patil & Gaikwad [185]: 22862; Saha et al. [27]: 20; Bajracharya & Budha [99]: 853; Ye et al. [75]: 351.

**Distribution. Oriental.** China (Taiwan, Yunnan, Guizhou, Guangxi, Hainan); - Bhutan, India, Nepal, Thailand, Indonesia (Java), Philippines, Vietnam [1,99,107].
**Host plants. Cucurbitaceae** [22,161], *Benincasa hispida*, *Citrullus lanatus*, *Cucurbita moschata*, *Cucumis sativus* [23], *Cuccinia indica* [107], *Coccinia grandis* [16], *Gymnopetalum chinense*, *Luffa aegytiaca*, *L*. *acutangula*, *Sechium edule* [23], *Trichosanthes bracteata* [16]; **Fabaceae:** *Phaseolus vulgaris* [16], **Solanaceae:** *Solanum lycopersicum*, *S. melongena*, *S. tuberosum* [16,27], *S. nigrum*, *S. verbascifolium* [16].

135) ***Henosepilachna pytharga* (Dieke, 1947)**

*Epilachna pytharga* Dieke, 1947 [114]: 74. Type locality: Philippine Islands. — Bielawski [120]: 383.

*Henosepilachna pytharga* (Dieke [114]): — Pang [58]: 106; Yu & Pang [118]: 14; Jadwiszczak & Węgrzynowicz [1]: 193; Kovář [94]: 629; Nakano [16]: 162; Ye et al. [75]: 351.

**Distribution. Oriental.** China (Taiwan); - Philippines (Bomeo, Sumatra) [1,58,75].

136) ***Henosepilachna quadriplagiata* Pang & Mao, 1977**

*Henosepilachna quadriplagiata* Pang & Mao, 1977 [52]: 327, 328. Type locality: China (Xizang). — Li [121]: 401; Jadwiszczak & Węgrzynowicz [1]: 166; Kovář [94]: 629; Nakano [16]: 162; Ye et al. [75]: 352.

**Distribution. Palaearctic**. China (Tibet) [1,75].

137) ***Henosepilachna septima* (Dieke, 1947)**

*Epilachna septima* Dieke, 1947 [114]: 58. Type locality: Vietnam. — Kapur [85]: 151; Katakura et al. [23]: 330, 333.

*Epilachna keiseri* Bielawski, 1957b [129]: 73. Type locality: Sri Lanka. Synonymized by Kapur [85].

*Henosepilachna septima* (Dieke [114]): — Pang & Mao [53]: 110; Wang & Cao [92]: 1; Kobayashi et al. [161]: 148; Yu [189]: 240; Zeng [188]: 43; Chakraborty & Biswas [117]: 149; Jadwiszczak & Węgrzynowicz [1]: 170; Poorani [93]: 48; Kovář [94]: 629; Ren et al. [9]: 308; Canepari [15]: 367; Hayat & Khan [133]: 346; Katoh et al. [22]: 822; Saeed et al. [137]: 1371; Dorji et al. [26]: 518; Nakano [16]: 163; Patil & Gaikwad [185]: 22862; Saha et al. [27]: 20; Iqbal et al. [20]: 86; Bajracharya & Budha [99]: 853; Ye et al. [75]: 353.

**Distribution. Oriental.** China (Yunnan, Guizhou, Guangdong, Guangxi, Hainan); - Bhutan, India, Sri Lanka, Pakistan, Vietnam [1,9,16,20,75].
**Host plants. Cucurbitaceae** [22,27,161], *Benincasa hispida* var. *chiehqua* [16], *Momordica charantia* [16,20,23], *M. subangulata* [23], *Luffa cylindrica* [20].

138) ***Henosepilachna sexta* (Dieke, 1947)**

*Epilachna sexta* Dieke, 1947 [114]: 84. Type locality: Indonesia (Celebes, Molino).

*Henosepilachna sexta* (Dieke [114]): — Jadwiszczak & Węgrzynowicz [1]: 170; Nakano [16]: 163; Ye et al. [75]: 354.

**Distribution. Oriental.** China (Yunnan); - Indonesia [1,16,75].
**Host plant. Solanaceae**: *Solanum nigrum* [16].

139) ***Henosepilachna subfasciata* (Weise, 1923)**

*Epilachna subfasciata* Weise, 1923b [44]: 182. Type locality: China (Taiwan).

*Epilachna semifasciata* Dieke, 1947 [114]: 103. Type locality: China (Taiwan). Synonymized by Bielawski [50]: 213.

*Henosepilachna semifasciata* (Dieke [114]): — Li & Cook [21]: 50; Pang [58]: 105; Yu & Pang [118]: 14.

*Henosepilachna subfasciata* (Weise [44]): — Bielawski [50]: 213; Pang [58]: 105, 106; Yu & Pang [118]: 14; Jadwiszczak & Węgrzynowicz [1]: 172; Kovář [94]: 630; Nakano [16]: 163; Ye et al. [75]: 355.

**Distribution. Oriental.** China (Taiwan) [1,50,58,75].
**Host plants. Solanaceae:** *Lycianthes bifiora*, *Solanum nigrum* [16].

140) ***Henosepilachna tamdaoensis* Hoàng, 1977**

*Henosepilachna tamdaoensis* Hoàng, 1977 [87]: 137. Type locality: Vietnam. — Zeng [65]: 139; Pang & Zeng [67]: 283; Jadwiszczak & Węgrzynowicz [1]: 174; Kovář [94]: 630; Ren et al. [9]: 308; Zhang & Ou [109]: 57; Nakano [16]: 164; Ye et al. [75]: 356.

**Distribution. Oriental.** China (Yunnan, Guizhou, Guangdong, Guangxi); - Vietnam [1,16,75].

141) ***Henosepilachna tenuis* Ye, Chen & Wang, 2026**

*Henosepilachna tenuis* Ye, Chen & Wang *in* Ye et al. 2026 [75]: 357. Type locality: China (Xizang).

**Distribution. Palaearctic.** China (Xizang) [75].

142) ***Henosepilachna tonkinensis* (Bielawski, 1957)**

*Epilachna tonkinensis* Bielawski, 1957a [191]: 91. Type locality: Vietnam.

*Henosepilachna tonkinensis* (Bielawski [191]): — Jadwiszczak & Węgrzynowicz [1]: 175; Ren et al. [9]: 310; Zhang & Ou [109]: 57; Tomaszewska & Szawaryn [2]: 56; Nakano [16]: 164; Ye et al. [75]: 358.

**Distribution. Palaearctic.** China (Tibet); - **Oriental.** China (Yunnan, Guizhou, Guangxi); - Vietnam [1,16,75].

143) ***Henosepilachna umbonata* Pang & Mao, 1979**

*Henosepilachna umbonata* Pang & Mao, 1979 [53]: 118. Type locality: China (Yunnan). — Jadwiszczak & Węgrzynowicz [1]: 176; Kovář [94]: 630; Ren et al. [9]: 310; Nakano [16]: 164; Ye et al. [75]: 359.

**Distribution. Oriental**. China (Yunnan) [1,75].

144) ***Henosepilachna verriculata* Pang & Mao, 1979**

*Henosepilachna verriculata* Pang & Mao, 1979 [53]: 117. Type locality: China (Yunnan). — Jadwiszczak & Węgrzynowicz [1]: 177; Kovář [94]: 630; Ren et al. [9]: 310; Zhang & Ou [109]: 57; Nakano [16]: 164; Poorani & Thangjam [140]: 557; Chandra et al. [159]: 417; Iqbal et al. [20]: 87; Ye et al. [75]: 360

**Distribution. Oriental.** China (Yunnan); - Pakistan, India [1,20,75,140].
**Host plant. Cucurbitaceae:** *Solena amplexicaulis* [20].

145) ***Henosepilachna vigintioctomaculata* (Motschulsky, 1857)**

*Epilachna 28-maculata* Motschulsky, 1857 [192]: 40. Type locality: Japan.

*Epilachna 28-maculata* a. *incompleta* Mader, 1930 [45]: 184. Type locality: Korea.

*Epilachna 28-maculata* a. *coalescens* Mader, 1930 [45]: 184. Type locality: China (Sichuan). — Liu [48]: 164.

*Epilachna vigintioctomaculata* Motschulsky [192]: — Liu [48]: 164; Bielawski [178]: 436; Kobayashi et al. [161]: 148.

*Epilachna vigintioctomaculata solanivora* Chujo, 1968 [193]: 52. *Nomen nudum*.

*Henosepilachna vigintioctomaculata* (Motschulsky [192]): — Li & Cook [21]: 48; Pang & Mao [52]: 324; Pang & Mao [53]: 111; Li [121]: 400; Xiao & Li [57]: 384; Canepari [132]: 56; Yu & Pang [118]: 14; Zhang & Yu [162]: 430; Yu [189]: 239; Kuznetsov & Zakharov [144]: 173; Jadwiszczak & Węgrzynowicz [1]: 178; Poorani [93]: 48, 49; Kovář [94]: 630; Ren et al. [9]: 312; Zhang & Ou [109]: 56; Katoh et al. [22]: 822; Tomaszewska & Szawaryn [2]: 56; Szawaryn [150]: 161; Cho [145]: 311; Dorji et al. [26]: 518; Nakano [16]: 164; Das et al. [35]: 41; Yu [163]: 192; Huang et al. [12]: 19; Bajracharya & Budha [99]: 854; Ye et al. [75]: 361.

**Distribution. Australian.** Australia; **Oriental.** China (Yunnan, Guizhou, Sichuan, Hubei, Jiangsu, Zhejiang, Guangxi, Taiwan); - Bhutan, India, Nepal, Vietnam; **Palaearctic.** China (Beijing, Gansu, Hebei, Heilongjiang, Henan, Jilin, Liaoning, Shanxi, Shaanxi, Shandong, Tibet); –Japan, Korea, Russian Far East (Primorskij Kraj, Khabarowskij Kraj) [1,16,75,144,163].
**Host plants. Cucurbitaceae** [22,161], *Cucumis sativus*, *Cucurbita pepo*, *C. moschata*, *Schizopepon bryoniifolius* [16]; **Fabaceae:** *Phaseolus vulgaris* [16]; **Schisandraceae:** *Schisandra* cf. *Propinqua* [16]; **Solanaceae** [22,161,163], *Capsicum annuum* [16]; *Datura stramonium* [16,109], *D. metel* [16], *Lycopersicon esculentum* [16,109], *Lycium barbarum*, *L. chinense* [16], *Solanum melongena*, *S. nigrum*, *S. tuberosum* [16,109].

146) ***Henosepilachna vigintioctopunctata* (Fabricius, 1775)**

*Coccinella 28 punctata* Fabricius, 1775 [165]: 84. Type locality: India.

*Coccinella chrysomelina* Fabricius, 1775 [165]: 82. Type locality: Saint Helena. Synonymized by Fürsch [78].

*Coccinella sparsa* Herbst, 1786 [180]: 160. Type locality: Ostindien. Synonymized by Herbst, 1793 [194].

*Coccinella 24maculata* Fabricius, 1792 [195]: 281. Type locality: India. Synonymized by Mulsant [128].

*Coccinella pubescens* Hope, 1831 [155]: 31. Type locality: Nepal. Synonymized by Mulsant [128].

*Coccinella pardalis* Boisduval, 1835 [196]: 596. Type locality: Santa Cruz Island (Vanikoro). Synonymized by Korschefsky [76]: 26.

*Coccinella 11-variolata* Boisduval, 1835 [196]: 590. Type locality: New Guinea.

*Epilachna infausta* Mulsant, 1850 [128]: 786. Type locality: Indonesia (Java). Synonymized with *Coccinella sparsa* Herbst, 1786 [180] by Fürsch [77].

*Epilachna territa* Mulsant, 1850 [128]: 787. Type locality: Indonesia (Java). Synonymized by Richards, 1983 [197]: 18.

*Epilachna territa* var. *indocilis* Mulsant, 1850 [128]: 788. Type locality: Maluku.

*Epilachna territa* var. *fatalis* Mulsant, 1850 [128]: 788. Type locality: Not available.

*Epilachna territa* var. *lusoria* Mulsant, 1850 [128]: 789. Type locality: Not available.

*Epilachna gradaria* Mulsant, 1850 [128]: 789. Type locality: India. Synonymized with *Coccinella sparsa* Herbst, 1786 [180] by Dieke [114]: 33.

*Epilachna gradaria* var. *addita* Mulsant, 1850 [128]: 791. Type locality: India.

*Epilachna gradaria* var. *socors* Mulsant, 1850 [128]: 791. Type locality: Indes Orientales.

*Epilachna gradaria* var. *stolida* Mulsant, 1850 [128]: 791. Type locality: Indonesia (Java).

*Epilachna gradaria* var. *vieta* Mulsant, 1850 [128]: 791. Type locality: Not available.

*Epilachna vigintiocto-punctata* var. *egens* Mulsant, 1850 [128]: 836. Type locality: Indonesia (Java).

*Epilachna vigintiocto-punctata* var. *multi-punctata* Mulsant, 1850 [128]: 836. Type locality: Not available.

*Epilachna vigintiocto-punctata* var. *recta* Mulsant, 1850 [128]: 836. Type locality: Not available.

*Epilachna implicata* var. *lacertosa* Mulsant, 1850 [128]: 838. Type locality: Indes Orientales. Synonymized by Crotch [38].

*Coccinella* (*Epilachna*) *28-punctata* Montrouzier, 1857 [198]: 75, nee *Coccinella 28 punetata* Fabricius, 1775 [165]. Type locality: Woodlark. Synonymized by Jadwiszczak & Węgrzynowicz [1]. Montrouzier’s *Coccinella 28-punctata* is a junior primary homonym of Fabricius’ name; being also a junior synonym it does not warrant creation of a replacement-name.

*Epilachna gradaria* var. *infuscata* Weise, 1895 [199]: 152. Type locality: Barway.

*Epilachna 28-punctata* a. *imitata* Mader, 1927a [200]: 35. Type locality: Not available.

*Epilachna sparsa orientalis* Dieke, 1947 [114]: 34, nee *Epilachna elaterii orientalis* Zimmermann, 1934 [47]: 527. Type locality: China. Despite homonymy of Dieke’s name, creation of a replacement-name is not warranted because of uncertain Status of this subspecies and chaos in the systematics of this species group.

*Epilachna sparsa orientalis* var. *cinerea* Dieke, 1947 [114]: 35. Type locality: China (Sichuan). Unavailable name in zoological nomenclature (ICZN, Art. 5).

*Epilachna sparsa 26-punctata* var. *nigrescens* Dieke, 1947 [114]: 37. Type locality: Fiji. Unavailable name in zoological nomenclature (ICZN, Art. 5).

*Epilachna vigintioctopunetata* ab. *nakana* Nakane & Araki, 1959 [184]: A45. Type locality: Japan.

*Epilachna 28-punctata* Fabricius [165]: — Kapur [82]: 310; Booth & Pope [152]: 346.

*Epilachna vigintioctopunctata* (Fabricius [165]): — Kapur [85]: 151; Kapur [86]: 310; Canepari [135]: 29; Abbas et al. [201]: 45; Shirai & Katakura [107]: 76; Katakura et al. [23]: 327; Joshi & Sharma [202] 2008: 163; Casaria & Teixeira [111]: 114.

*Henosepilachna vigintioctopunctata* (Fabricius [165]): — Pang & Mao [53]: 108; Miyatake [89]: 22; Xiao & Li [57]: 384; Li [149]: 211; Pang [58]: 105; Yu & Pang [118]: 14; Zhang & Yu [162]: 430; Canepari [132]: 56; Kobayashi et al. [161]: 148; Yu [189]: 240; Yu & Lau [108]: 171; Pang & Zeng [67]: 283; Chakraborty & Biswas [117]: 149; Jadwiszczak & Węgrzynowicz [1]: 178; Poorani [93]: 49; Kovář [94]: 630; Ślipiński [31]: 544; Joshi & Sharma [202]: 163; Ren et al. [9]: 312; Zhang & Ou [109]: 56; Canepari [15]: 367; Naz et al. [110]: 421; Hayat & Khan [133]: 346; Katoh et al. [22]: 822; Bhatnagar [203]: 81; Saeed et al. [137]: 1371; Tomaszewska & Szawaryn [2]: 56; Ahmed et al. [112]: 1754; Halim et al. [187]: 815; Sajan et al. [25]: 1244; Szawaryn [150]: 161; Cho [145]: 311; Dorji et al. [26]: 518; Nakano [16]: 165; Nakano [204]: 138; Sajan et al. [205]: 424; Yu [163]: 192; Patil & Gaikwad [185]: 22862; Saha et al. [27]: 20; Huang et al. [12]: 19; Iqbal et al. [20]: 82; Bajracharya & Budha [99]: 853; Duarte-de-Mélo et al. [17]: 470; Ye et al. [75]: 362.

**Distribution. Oriental.** China (Anhui, Chongqing, Hubei, Fujian, Guangdong, Guangxi, Guizhou, Hainan, Hong Kong, Hunan, Jiangsu, Jiangxi, Tibet, Sichuan, Taiwan, Yunnan, Zhejiang); - Bhutan, India, Malaysia, Myanmar, Thailand, Vietnam, Philippine, Indonesia (Sumatra, Java), Pakistan, Nepal, Sri Lanka; **Palaearctic.** China (Beijing, Hebei, Henan, Liaoning, Shandong, Shanxi, Shaanxi); - Afghanistan, Japan, Korea; **Australian.** Australia, New Guinea, Fiji, Solomon Is.; **Neotropical.** Brazil [1,15,16,17,58,75,107].
**Host plants. Adoxaceae:** *Sambucus racemosa* subsp. *sieboldiana*; **Apocynaceae:** *Calotropis procera* [20]; **Araliaceae:** *Panax japonicus* [204]; **Asteraceae:** *Arctium lappa* [204], *Bidens pilosa* [16,109], *Carduus crispus* [204], *Chromolaena odorata* [23], *Cirsium kamtschaticum*, *C. setosum* [204], *Erigeron canadensis*, *Parthenium hysterophorus* [20]; **Boraginaceae:** *Symphytum tuberosum* [112]; **Brassicaceae:** *Brassica chinensis* [108,204], *B. rapa* var. *glabra*, *B. rapa* var. *hakabura*, *B. rapa* var. *rapa*, *Raphanus sativus* var. *hortensis* [204], *Raphanus sativus* [108]; **Convolvulaceae:** *Ipomoea cairica* [108]; **Cucurbitaceae** [161], *Actinostemma tenerum* [204], *Benincasa hispida* [108], *Citrullus lanatus* [204], *Cucumis melo* [204], *C. sativus* [16,108,204], *Cucurbita maxima* [204], *C. moschata* [20,204], *Lagenaria siceraria* [20], *Luffa aegyptica* [204], *L. cylindrica* [16,108], *Momordica charantia* [108], *Trichosanthes kirilowii* var. *japonica* [204]; **Dennstaedtiaceae:** *Pteridium aquilinum* [204]; **Elaeagnaceae:** *Elaeagnus multiflora* [204]; **Fabaceae** [22], *Centrosema pubescens* [23,107], *Glycine soja* [16], *G. max*, *Phaseolus coccineus* [204], *P. mungo* [108], *P. vulgaris* [204], *Trifolium alexandrinum* [20], *Vicia faba*, *Vigna angularis* var. *angularis*, *V. unguiculata* var. *Unguiculata* [204]; **Juncaceae:** *Juncus compressus* [20]; **Lythraceae:** *Lagerstroemia indica* [20]; **Poaceae:** *Saccharum spontaneum*, *Tripidium bengalense* [20], *Zea mays* [108]; **Polygonaceae:** *Fallopia sachalinensis* [204]; **Portulacaceae:** *Portulaca oleracea* [204]; **Solanaceae** [22,161], *Alkekengi officinarum* var. *franchetii* [204], *Brugmansia candida*, *B. suaveolens* [23,111], *Capsicum annuum* [16,204], *C. frutescens* [108], *Datura metel* [23,108,201], *D. stramonium* [16,109,204], *Lycopersicon esculentum* [16,23,108,109,110], *Lycium barbarum* [16], *Lycium chinense* [108,204], *L. cylindrica* [108], *Nicotiana rustica* [204], *N. tabacum* [108], *Petunia hybrida* [204], *Physaliastrum echinatum* [204], *Physalis* sp. [110], *P. angulata* [108], *P. heterophylla* [204], *P. peruviana* [23], *Withania somnifera* [20,110], *Scopolia japonica* [204], *Solanum aethiopicum*, *S. americanum*, *S. capsicoides* [23], *S. carolinense* [16,109], *S. erianthum* [23], *S. indicum* [16,108,109,204], *S. jamaicaense* [23], *S. japonense* [204], *S. lycopersicum* [27,204], *S. macrocarpon* [23], *S. mammosum* [23,108], *S. maximowiczii* [204], *S. melongena* [16,20,23,27,107,108,109,110,201,204], *S. nigrum* [16,20,23,107,108,109,110], *S. pseudocapsicum* [23,107], *S. surretanses* [110], *S. torvum* [16,23,107,109,201], *S. triflorum* [23]; *S. tuberosum* [16,23,27,107,108,109,201]; **Urticaceae:** *Urtica platyphylla* [204].

147) ***Henosepilachna wissmanni* (Mulsant, 1850)**

*Epilachna wissmanni* Mulsant, 1850 [128]: 832. Type locality: Celebes.

*Epilachna wissmanni* ab. *forsteri* Weise, 1908 [190]: 218. Type locality: Sumatra.

*Henosepilachna wissmani* (Mulsant [128]): — an incorrect spelling used by Nakano [16]: 166.

*Henosepilachna wissmanni* (Mulsant [128]): — Yu & Pang [118]: 14; Jadwiszczak & Węgrzynowicz [1]: 182; Katoh et al. [22]: 822; Ye et al. [75]: 364.

**Distribution. Oriental.** China (Taiwan); - India, Thailand, Philippines, Indonesia (Borneo, Celebes, Sumatra) [1,16,75].
**Host plant. Cucurbitaceae** [22].
**Genus *Subcoccinella* Agassiz & Erichson, 1845**

*Lasia* Hope, 1840 [166]: 157. (nec Wiedemann, 1824 [206]: 11; Diptera). Type species: *Coccinella globosa* Schneider, 1792 [207] (=*Coccinella vigintiquatuorpunctata* Linnaeus, 1758 [208]). Original designation.

*Subcoccinella* Agassiz & Erichson, 1845 [209]. Type species: *Coccinella vigintiquatuorpunctata* Linnaeus, 1758 [208]. Replacement name for *Lasia* Hope, 1840 [166]: 157. — Pang & Mao [53]: 160; Fürsch [79]: 397; Jadwiszczak & Węgrzynowicz [1]: 184; Kovář [94]: 630; Ren et al. [9]: 314; Szawaryn et al. [34]: 566; Tomaszewska & Szawaryn [2]: 68; Cho [145]: 311; Nakano [16]: 166.

**Remarks.** The genus *Subcoccinella* comprises two described species, both endemic to the Palaearctic region. *Subcoccinella vigintiquatuorpunctata* is the only species of the genus recorded from China and is also widely distributed across the Palaearctic region. It has been recorded on *Medicago* sp. (family Fabaceae).

148) ***Subcoccinella vigintiquatuorpunctata* (Linnaeus, 1758)**

*Coccinella 24-punctata* Linnaeus, 1758 [208]: 366. Type locality: Europa.

*Coccinella 25-punctata* Linnaeus, 1758 [208]: 366. Type locality: Europa. Synonymized by Paykull, 1798 [210].

*Coccinella haemorrhoidalis* Fabricius, 1776 [211]: 218. Type locality: Germany (Hamburg). Synonymized by Herbst, 1793 [194].

*Coccinella livida* Herbst, 1783 [212]: 42. Type locality: Germany (Berlin). Synonymized by Herbst, 1793 [194].

*Coccinella colon* Herbst, 1783 [212]: 42. Type locality: Germany (Berlin). Synonymized by Herbst, 1793 [194].

*Coccinella limbata* Moll, 1784 [213]: 181, nee Fabricius [214]: 497. Type locality: Austria (Salzburg). Synonymized by Weise *in* Heyden, Reitter, Weise [215].

*Coccinella 4 notata* Fabricius, 1787 [216]: 56. Type locality: Kiliae. Synonymized by Schönherr, 1808 [127]: 154.

*Coccinella globosa* Schneider, 1792 [207]: 149. Type locality: Europa. Synonymized by Illiger, 1798 [217].

*Coccinella immaculata* Rossi, 1794 [218]: 86, nee Gmelin, 1790: 1644, nee Fabricius [195]: 267. Type locality: Not available. Synonymized by Gemminger & Harold, 1876 [219].

*Coccinella hemisphaerica* Schrank, 1798 [220]: 460. Type locality: Baiern. Note: Schrank, 1798 [220] included name *Coccinella 24-punctata* Linnaeus, 1758 [208] in the list of Synonyms of *Coccinella hemisphaerica*, so it is younger objective synonym of Linnaeus’ name.

*Coccinella 24-punctata* var. *22-punctata* Haworth, 1812 [221]: 283. Type locality: Not available.

*Coccinella 24-punctata* var. *vulgaris* Haworth, 1812 [221]: 283. Type locality: Not available.

*Coccinella rufet* Haworth, 1812 [221]: 283. Type locality: Not available. Synonymized by Crotch [38].

*Coccinella rufa* var. *22-punctata* Haworth, 1812 [221]: 284. Type locality: Not available.

*Coccinella rufa* var. *20-punctata* Haworth, 1812 [221]: 284. Type locality: Not available.

*Coccinella gibbosa* Dumeril, 1817 [222]: 495. Type locality: Not available. Synonymized by Mulsant, 1846 [223].

*Coccinella meridionalis* Motschulsky, 1837 [224]: 420. Type locality: Derbent and in the provinces bordering the Caspian Sea. Synonymized by Crotch [38].

*Coccinella colchica* Motschulsky, 1839 [225]: 51. Type locality: Republic of Georgia. Synonymized by Crotch [38].

*Subcoccinella vigintiquatuorpunctata* v. *saponariae* Weise, 1879 [226]: 130. Type locality: Not available. Note: Hitherto *Subcoccinella saponariae* has been attributed to Huber, 1842 [227]: 353 who, however, used only a vernacular name; the first to introduce *saponariae* was Weise [226].

*Subcoccinella 24-punctata* var. *centrimaculata* Rossi, 1882 [228]: 214. Type locality: Not available.

*Subcoccinella 24-punctata* var. *zonata* Heyden, 1883 [229]: 53. Type locality: Not available.

*Subcoccinella 24-punctata* var. *inversa* Weise, 1905b [230]: 137. Type locality: Germany (Berlin).

*Subcoccinella vigintiquatuor-punctata* var. *reticulata* Della-Beffa, 1912 [231]: 184. Type locality: Italia.

*Subcoccinella vigintiquatuor-punctata* var. *reticulata* ab. *bifasciata* Della-Beffa, 1912 [231]: 185. Note: Infrasubspecific *quadrinomen*, unavailable in zoological nomenclature (ICNZ, Art. 5).

*Subcoccinella vigintiquatuor-punctata* var. *reticulata* ab. *laterifasciata* Della-Beffa, 1912 [231]: 185. Note: Infrasubspecific *quadrinomen*, unavailable in zoological nomenclature (ICNZ, Art. 5).

*Subcoccinella vigintiquatuor-punctata* var. *reticulata* ab. *festae* Della-Beffa, 1912 [231]: 186. Note: Infrasubspecific *quadrinomen*, unavailable in zoological nomenclature (ICNZ, Art. 5).

*Subcoccinella vigintiquatuor-punctata* var. *nigra* Fiori in Della-Beffa, 1912 [231]: 187. Type locality: Bologna.

*Subcoccinella* (*Lasia*) *24-punctata* v. *biundulata* Pic, 1912 [232]: 82. Type locality: Cancale.

*Subcoccinella 24-punctata* ab. *nigra* Hänel, 1913 [233]: 99. Type locality: bei Klausen in Südtirol.

*Subcoccinella 24-punctata* a. *parvimacula* Depoli, 1915 [234]:109. Type locality: Martinschizza bei Fiume.

*Subcoccinella 24-punctata forma micropunctata* Papp, 1943 [235]: 195. Type locality: Not available.

*Subcoccinella 24-punctata forma hungarica* Papp, 1943 [235]: 195. Type locality: Not available.

*Subcoccinella 24-punctata forma marmorata* Papp, 1943 [235]: 195. Type locality: Not available.

*Subcoccinella 24-punctata ab. quadriundulata* Krejcärek, 1949 [236]: 118. Type locality: Bzenec.

*Subcoccinella 24-punctata* ab. *triundulata* Krejcärek, 1949 [236]: 118. Type locality: Uh. Hradiste.

*Subcoccinella vigintiquatuorpunctata* (Linnaeus [208]): — Bielawski, 1968 [237]: 193; Bielawski, 1975 [238]: 247; Fürsch [79]: 397; Kuznetsov & Zakharov [144]: 173; Jadwiszczak & Węgrzynowicz [1]: 184; Kovář [94]: 630; Ren et al. [9]: 314; Magro et al. [239]: 835; Tomaszewska & Szawaryn [2]: 71; Cho [145]: 311; Nakano [16]: 167; Soares et al. [240]: 122; Sanchez & Chittaro [241]: 116; Biranvand et al. [19]: 111.

**Distribution. Palaearctic.** China (Liaoning, Xinjiang); - widely distributed [1,9,241,242].
**Host plant. Fabaceae:** *Medicago* sp. [16].
**Genus *Uniparodentata* Wang & Cao, 1993**

*Epilachna* (*Uniparodentata*) Wang & Cao, 1993 [91]: 126. Type species: *Epilachna paramagna* Pang & Mao [53]: 135. Original designation.

*Epilachna* (*Aparodentata*) Wang & Cao, 1993 [91]: 126. Type species: *Epilachna yongshanensis* Cao & Xiao [55]: 116, 128. Original designation. Synonymized by Tomaszewska & Szawaryn [2]: 40.

*Ryszardia* Tomaszewska & Szawaryn *in* Szawaryn et al. [34]: 563. Type species: *Epilachna decipiens* Crotch, 1874 [38]: 83. Original designation). Synonymized by Tomaszewska & Szawaryn [2]: 40.

*Uniparodentata* Wang & Cao [91]: — Das et al. [35]: 252; Tomaszewska & Szawaryn [2]: 39; Bajracharya & Budha [99]: 854; Iqbal et al. [20]: 58.

**Remarks.** The genus *Uniparodentata* currently includes 34 species, predominantly distributed in the Oriental region, with 6 species also extending into the Palaearctic. The Chinese fauna comprises 28 species, 26 of the which are endemic to China, while the other 2 species are also recorded from India and Korea. Among the endemic species, 7 species are known only in Taiwan, followed by Yunnan (5), Sichuan (4), Guizhou (1), and Hunan (1). The provisional regional distribution records by region are as follows: Sichuan (9), Taiwan (9), Yunnan (8), Guizhou (7), Guangdong (5), Fujian (3), Guangxi (3), Hunan (3), Shaanxi (3), Shandong (2), Jiangsu (2), and a single species each from Gansu, Hainan, Henan, Hong Kong, Hubei, Jiangxi, Tibet, and Zhejiang. Host plant records are available for 13 species, revealing associations with 20 plant species under nine families. Ranunculaceae is the most frequently recorded host family (5 species), followed by Schisandraceae (4), Solanaceae (4), Oleaceae (3), and Cucurbitaceae (2). Lamiaceae, Rosaceae, Rubiaceae, and Urticaceae are each represented by a single species.

149) ***Uniparodentata acuta* (Weise, 1900)**

*Epilachna acuminata* Weise, 1889 [39]: 648. Type locality: China (Gansu). Preoccupied by *Epilachna acuminata* Mulsant, 1853 [37]: 240.

*Solanophila acuta* Weise, 1900b [243]: 384. Replacement name for *Epilachna acuminata* Weise, 1889 [39]: 648.

*Solanophila acutula* Weise, 1902b [174]: 496. An unnecessary replacement name for *Solanophila acuminata* (Weise, 1889 [39]: 648).

*Epilachna acuta* (Weise [174]): — Li & Cook [21]: 89; Bielawski, 1965a [244]: 217; Jadwiszczak & Węgrzynowicz [1]: 32; Kovář [94]: 626.

*Ryszardia acuta* (Weise [39]): — Szawaryn et al. [34]: 563.

*Uniparodentata acuta* (Weise [39]): — Tomaszewska & Szawaryn [2]: 42; Nakano [16]: 139.

**Distribution. Oriental.** China (Hubei, Jiangsu, Taiwan); **Palaearctic.** China (Gansu, Henan, Shaanxi) [1,69,134].

150) ***Uniparodentata angusta* (Li, 1961)**

*Epilachna angusta* Li *in* Li & Cook, 1961 [21]: 79. Type locality: China (Taiwan). — Pang & Mao [53]: 146; Pang [58]: 106; Yu & Pang [118]: 14; Jadwiszczak & Węgrzynowicz [1]: 35; Kovář [94]: 626; Ren et al. [9]: 262; Pang et al. [69]: 34.

*Ryszardia angusta* (Li *in* Li & Cook [21]): — Szawaryn et al. [34]: 563.

*Uniparodentata angusta* (Li *in* Li & Cook [21]): — Tomaszewska & Szawaryn [2]: 42; Nakano [16]: 139.

**Distribution. Oriental**. China (Taiwan) [1,21,58,69].
**Host plant. Rosaceae:** *Rubus lambertianus* [16].

151) ***Uniparodentata bifibra* (Li, 1961)**

*Epilachna bifibra* Li *in* Li & Cook, 1961 [21]: 82. Type locality: China (Taiwan). — Yu & Pang [118]: 14; Jadwiszczak & Węgrzynowicz [1]: 40; Kovář [94]: 626.

*Ryszardia bifibra* (Li *in* Li & Cook [21]): — Szawaryn et al. [34]: 563.

*Uniparodentata bifibra* (Li *in* Li & Cook [21]): — Tomaszewska & Szawaryn [2]: 42; Nakano [16]: 140.

**Distribution. Oriental.** China (Taiwan) [1,2,21].

152) ***Uniparodentata boymi* (Jadwiszczak & Węgrzynowicz, 2003)**

*Solanophila hauseri* Mader, 1930 [45]: 182. Preoccupied by *Epilachna hauseri* Weise, 1904a: 57. Type locality: China (Yunnan).

*Epilachna boymi* Jadwiszczak & Węgrzynowicz, 2003 [1]: 45. A new replacement name for *Epilachna hauseri* (Mader, 1930 [45]: 182). — Wang & Cao [91]: 120; Wang & Cao [122]: 117; Jadwiszczak & Węgrzynowicz [1]: 45.

*Uniparodentata boymi* (Jadwiszczak & Węgrzynowicz): — Tomaszewska & Szawaryn [2]: 42; Nakano [16]: 141.

**Distribution. Oriental.** China (Guizhou, Sichuan, Yunnan); **Palaearctic.** China (Shandong) [1,2,69].
**Host plants. Ranunculaceae:** *Clematis armandii*, *C. fasciculifolata* [16].

153) ***Uniparodentata chingjing* (Yu & Wang, 1999)**

*Epilachna chingjing* Yu & Wang, 1999 [62]: 28. Type locality: China (Taiwan). — Jadwiszczak & Węgrzynowicz [1]: 49; Kovář [94]: 627; Pang et al. [69]: 34.

*Ryszardia chingjing* (Yu & Wang [62]): — Szawaryn et al. [34]: 563.

*Uniparodentata chingjing* (Yu & Wang [62]): — Tomaszewska & Szawaryn [2]: 43; Nakano [16]: 142.

**Distribution. Oriental**. China (Taiwan) [1,2,62,69].
**Host plant. Cucurbitaceae:** *Thladiantha nudiflora* [16].

154) ***Uniparodentata circummaculata* (Pang & Mao, 1977)**

*Epilachna circummaculata* Pang & Mao, 1977 [52]: 325. Type locality: China (Tibet). — Pang & Mao [53]: 141; Li [121]: 399; Jadwiszczak & Węgrzynowicz [1]: 45; Kovář [94]: 627; Ren et al. [9]: 268; Pang et al. [69]: 34; Das et al. [35]: 252.

*Ryszardia circummaculata* (Pang & Mao [52]): — Szawaryn et al. [34]: 563.

*Uniparodentata circummaculata* (Pang & Mao [52]): — Tomaszewska & Szawaryn [2]: 42; Nakano [16]: 143.

**Distribution. Oriental.** India; **Palaearctic.** China (Tibet) [1,35,52,69,134,139].

155) ***Uniparodentata clematicola* (Cao & Xiao, 1984)**

*Epilachna clematicola* Cao & Xiao, 1984 [55]: 115, 127. Type locality: China (Yunnan). — Cao [105]: 77, 82; Wang [245]: 193; Wang & Cao [91]: 120; Wang & Cao [122]: 118; Kovář [94]: 627; Zhang & Ou [109]: 53; Pang et al. [69]: 34.

*Epilachna clemeticola* Cao & Xiao [55]: — an incorrect spelling used by Jadwiszczak & Węgrzynowicz [1]: 50.

*Ryszardia clematicola* (Cao & Xiao [55]): — Szawaryn et al. [34]: 563.

*Uniparodentata clematicola* (Cao & Xiao [55]): — Tomaszewska & Szawaryn [2]: 43; Nakano [16]: 143.

**Distribution. Oriental.** China (Yunnan) [1,2,55,69,134].
**Host plants. Ranunculaceae:** *Clematis armandii* [16,105], *Clematis fasciculifiora* [16,109].

156) ***Uniparodentata complicata* (Dieke, 1947)**

*Afissa complicata* Dieke, 1947 [114]: 159. Type locality: China (Sichuan).

*Afissa complicata* var. *sustenans* Dieke, 1947 [114]: 161. China (Sichuan).

*Epilachna complicata* (Dieke [114]): — Jadwiszczak & Węgrzynowicz [1]: 51; Kovář [94]: 627; Pang et al. [69]: 34.

*Ryszardia complicata* (Dieke [114]): — Szawaryn et al. [34]: 563.

*Uniparodentata complicata* (Dieke [114]): — Tomaszewska & Szawaryn [2]: 43; Nakano [16]: 143.

**Distribution. Oriental**. China (Sichuan) [2,69,114,134].

157) ***Uniparodentata convexa* (Dieke, 1947)**

*Afissa convexa* Dieke, 1947 [114]: 158. Type locality: China (Sichuan). — Liu [116]: 28.

*Epilachna convexa* (Dieke [114]): — Pang & Mao [53]: 145; Xiao & Li [57]: 385; Jadwiszczak & Węgrzynowicz [1]: 53; Kovář [94]: 627; Ren et al. [9]: 270; Pang et al. [69]: 6, 34; Jamal et al. [246]: 406.

*Ryszardia convexa* (Dieke [114]): — Szawaryn et al. [34]: 563.

*Uniparodentata convexa* (Dieke [114]): — Tomaszewska & Szawaryn [2]: 43; Nakano [16]: 144.

**Distribution. Oriental.** China (Guizhou, Sichuan, Guangdong); **Palaearctic.** China (Shaanxi) [2,69,114,134].
**Host plant. Ranunculaceae:** *Anemone tomentosa* [16].

158) ***Uniparodentata crepida* (Pang & Ślipiński, 2012)**

*Epilachna crepida* Pang & Ślipińsk *in* Pang et al. 2012 [69]: 30. Type locality: China (Sichuan).

*Uniparodentata crepida* (Pang & Ślipiński *in* Pang et al. [69]): — Tomaszewska & Szawaryn [2]: 43; Nakano [16]: 144.

**Distribution. Oriental.** China (Sichuan) [2,69].

159) ***Uniparodentata exornata* (Bielawski, 1965)**

*Epilachna exornata* Bielawski, 1965c [50]: 223. Type locality: China (Yunnan). — Jadwiszczak & Węgrzynowicz [1]: 53; Kovář [94]: 627; Pang et al. [69]: 35.

*Ryszardia exornata* (Bielawski [50]): — Szawaryn et al. [34]: 563.

*Uniparodentata exornata* (Bielawski [50]): — Tomaszewska & Szawaryn [2]: 43; Nakano [16]: 145.

**Distribution. Oriental**. China (Yunnan) [2,50,69].

160) ***Uniparodentata folifera* (Pang & Mao, 1979)**

*Epilachna folifera* Pang & Mao, 1979 [53]: 149. Type locality: China (Yunnan). — Wang & Cao [91]: 120; Wang & Cao [122]: 117; Jadwiszczak & Węgrzynowicz [1]: 66; Kovář [94]: 627; Ren et al. [9]: 274; Zhang & Ou [109]: 56; Pang et al. [69]: 35.

*Ryszardia folifera* (Pang & Mao [53]): — Szawaryn et al. [34]: 563.

*Uniparodentata folifera* (Pang & Mao [53]): — Tomaszewska & Szawaryn [2]: 43; Nakano [16]: 146.

**Distribution. Oriental**. China (Yunnan) [1,2,69].

161) ***Uniparodentata fugongensis* (Cao & Xiao, 1984)**

*Epilachna fugongensis* Cao & Xiao, 1984 [55]: 111, 126. Type locality: China (Yunnan). — Wang & Cao [91]: 120; Wang & Cao [122]: 118; Jadwiszczak & Węgrzynowicz [1]: 67; Kovář [94]: 626; Pang et al. [69]: 35.

*Ryszardia fugongensis* (Cao & Xiao [55]): — Szawaryn et al. [34]: 563.

*Uniparodentata fugongensis* (Cao & Xiao [55]): — Tomaszewska & Szawaryn [2]: 43; Nakano [16]: 146.

**Distribution. Oriental**. China (Yunnan) [1,2,69].
**Host plant. Cucurbitaceae**: *Benincasa hispida* [16].

162) ***Uniparodentata glochisifoliata* (Pang & Mao, 1979)**

*Epilachna glochisifoliata* Pang & Mao, 1979 [53]: 139. Type locality: China (Sichuan). — Xiao & Li [57]: 385; Jadwiszczak & Węgrzynowicz [1]: 70; Kovář [94]: 626; Ren et al. [9]: 278; Zhang & Ou [109]: 55.

*Ryszardia glochisifoliata* (Pang & Mao [53]): — Szawaryn et al. [34]: 563.

*Uniparodentata glochisifoliata* (Pang & Mao [53]): — Tomaszewska & Szawaryn [2]: 43; Nakano [16]: 147.

**Distribution. Oriental.** China (Guangdong, Guangxi, Guizhou, Sichuan) [1,2,69].
**Host plants. Lamiaceae:** *Clerodendrum bungei*; **Urticaceae:***Urtica fissa* [109].

163) ***Uniparodentata gressitti* (Li, 1961)**

*Epilachna gressitti* Li *in* Li & Cook, 1961 [21]. 83. Type locality: China (Taiwan). — Yu & Pang [118]: 15; Jadwiszczak & Węgrzynowicz [1]: 72; Kovář [94]: 626; Pang et al. [69]: 35.

*Epilachna gressiti* Li *in* Li & Cook, 1961 [21]: — an incorrect spelling used by Szawaryn et al. [34]: 563.

*Uniparodentata gressiti* (Li *in* Li & Cook, 1961 [21]): — an incorrect spelling used by Tomaszewska & Szawaryn [2]: 1, 43.

*Ryszardia gressitti* (Li *in* Li & Cook, 1961 [21]): — Szawaryn et al. [34]: 563.

*Uniparodentata gressitti* (Li *in* Li & Cook, 1961 [21]): — Tomaszewska & Szawaryn [2]: 1, 43; Nakano [16]: 148.

**Distribution. Oriental.** China (Taiwan) [1,2,69].

164) ***Uniparodentata hamulifera* (Pang & Ślipiński, 2012)**

*Epilachna hamulifera* Pang & Ślipiński *in* Pang et al. 2012 [69]: 31, 35. Type locality: China (Hunan). — Nakano [16]: 148.

*Ryszardia hamulifera* (Pang & Ślipiński *in* Pang et al. [69]): — Szawaryn et al. [34]: 563.

*Uniparodentata hamulifera* Pang & Ślipiński *in* Pang et al. [69]: — Tomaszewska & Szawaryn [2]: 43.

**Distribution. Oriental.** China (Hunan) [2,69].

165) ***Uniparodentata lata* (Li, 1961)**

*Epilachna lata* Li *in* Li & Cook, 1961 [21]: 76. Type locality: China (Taiwan). — Bielawski [50]: 215; Yu & Pang [118]: 15; Jadwiszczak & Węgrzynowicz [1]: 82; Pang et al. [69]: 35.

*Ryszardia lata* (Li *in* Li & Cook, 1961): — Szawaryn et al. [34]: 563.

*Uniparodentata lata* (Li *in* Li & Cook, 1961): — Tomaszewska & Szawaryn [2]: 43; Nakano [16]: 150.

**Distribution. Oriental.** China (Taiwan) [1,69].

166) ***Uniparodentata madanensis* (Zeng, 2002)**

*Epilachna madanensis* Zeng *in* Pang & Zeng, 2002 [67]: 273. Type locality: China (Guizhou). — Ren et al. [9]: 286; Pang et al. [69]: 35; Nakano [16]: 151.

*Ryszardia madanensis* (Zeng *in* Pang & Zeng [67]): — Szawaryn et al. [34]: 563.

*Ryszardia madensis*: — an incorrect species name used by Szawaryn et al. [34]: 563.

*Uniparodentata madanensis* (Zeng *in* Pang & Zeng [67]): — Tomaszewska & Szawaryn [2]: 43.

**Distribution. Oriental.** China (Guizhou) [69].

167) ***Uniparodentata magna* (Dieke, 1947)**

*Afissa magna* Dieke, 1947 [114]: 167. Type locality: China (Fujian).

*Epilachna magna* (Dieke [114]): — Pang & Mao [53]: 143; Wang & Cao [91]: 120; Yu & Lau [108]: 171; Jadwiszczak & Węgrzynowicz [1]: 87; Kovář [94]: 628; Ren et al. [9]: 286; Zhang & Ou [109]: 55; Pang et al. [69]: 35; Nakano [16]: 151.

*Ryszardia magna* (Dieke [114]): — Szawaryn et al. [34]: 563.

*Uniparodentata magna* (Dieke [114]): — Tomaszewska & Szawaryn [2]: 43.

**Distribution. Oriental.** China (Fujian, Guangdong, Guizhou, Sichuan, Yunnan, Hong Kong) [69,108].
**Host plants. Solanaceae:** *Solanum melongena*, *S. tuberosum*, *S. verbascifolium* [16], *S. torvum* [16,109]; **Ranunculaceae:** *Clematis ganpiniana* [16,109].

168) ***Uniparodentata malleforma* (Peng, Pang & Ren, 2002)**

*Epilachna malleforma* Peng, Pang & Ren *in* Peng et al. 2002 [247]: 128, 130. Type locality: China (Hubei). — Kovář [94]: 628; Ren et al. [9]: 288; Pang et al. [69]: 13, 35; Nakano [16]: 151.

*Ryszardia malleforma* (Peng, Pang & Ren *in* Peng et al. [247]): — Szawaryn et al. [34]: 563.

*Uniparodentata malleforma* (Peng, Pang & Ren *in* Peng et al. [247]): — Tomaszewska & Szawaryn [2]: 43.

**Distribution. Oriental**. China (Hubei, Hunan); **Palaearctic**. China (Shaanxi) [69,247].

169) ***Uniparodentata media* (Li, 1961)**

*Epilachna media* Li *in* Li & Cook, 1961 [21]: 79. Type locality: China (Taiwan). — Yu & Pang [118]: 15; Jadwiszczak & Węgrzynowicz [1]: 89; Kovář [94]: 628; Pang et al. [69]: 35; Nakano [16]: 153.

*Ryszardia media* (Li *in* Li & Cook [21]): — Szawaryn et al. [34]: 563.

*Uniparodentata media* (Li *in* Li & Cook [21]): — Tomaszewska & Szawaryn [2]: 43.

**Distribution. Oriental**. China (Taiwan) [21,69].

170) ***Uniparodentata mobilitertiae* (Li, 1961)**

*Epilachna mobilitertiae* Li *in* Li & Cook, 1961 [21]: 83. Type locality: China (Taiwan). — Pang [58]: 107; Yu & Pang [118]: 15; Jadwiszczak & Węgrzynowicz [1]: 92; Kovář [94]: 628; Pang et al. [69]: 35.

*Uniparodentata mobliteratiae* (Li *in* Li & Cook [21]): — an incorrect spelling by Tomaszewska & Szawaryn [2]: 1, 43.

*Ryszardia mobilitertiae* (Li *in* Li & Cook [21]): — Szawaryn et al. [34]: 563.

*Uniparodentata mobilitertiae* (Li *in* Li & Cook [21]): — Tomaszewska & Szawaryn [2]: 43.

**Distribution. Oriental.** China (Taiwan) [21,58,69]^.^

171) ***Uniparodentata paramagna* (Pang & Mao, 1979)**

*Epilachna paramagna* Pang & Mao, 1979 [53]: 135. Type locality: China (Yunnan). — Wang & Cao [91]: 120; Wang & Cao [122]: 117; Jadwiszczak & Węgrzynowicz [1]: 102; Kovář [94]: 628; Ren et al. [9]: 294; Zhang & Ou [109]: 55; Pang et al. [69]: 36; Nakano [16]: 155.

*Uniparodentata paramagna* (Pang & Mao [53]): — Tomaszewska & Szawaryn [2]: 43.

**Distribution. Oriental.** China (Yunnan) [69].
**Host plant. Ranunculaceae:** *Clematis fasciculifiora* [109].

172) ***Uniparodentata quadricollis* (Dieke, 1947)**

*Afissa quadricollis* Dieke, 1947 [114]: 134. Type locality: China (Zhejiang).

*Epilachna quadricollis* (Dieke [114]): — Pang & Mao [53]: 149; Pang [58] 107; Yu & Pang [118]: 15; Zhang & Yu [162]: 430; Jadwiszczak & Węgrzynowicz [1]: 109; Kovář [94]: 628; Ren et al. [9]: 296; Pang et al. [69]: 22, 36; Cho [145]: 311; Nakano [16]: 156.

*Ryszardia quadricollis* (Dieke [114]): — Szawaryn et al. [34]: 563.

*Uniparodentata quadricollis* (Dieke [114]): — Tomaszewska & Szawaryn [2]: 43.

**Distribution. Oriental.** China (Fujian, Guangdong, Guangxi, Jiangsu, Jiangxi, Sichuan, Zhejiang, Taiwan); **Palaearctic.** China (Hebei, Shandong); - Korea [1,58,69].
**Host plants. Oleaceae:***Fraxinus chinensis*, *F. rhynchophylla*, *Ligustrum obtusifolium* [16].

173) ***Uniparodentata siphodenticulata* (Hoàng, 1983)**

*Epilachna siphodenticulata* Hoàng, 1983 [54]: 351. Type locality: China. — Zheng & Pang [143]: 119; Kovář [94]: 628; Ren et al. [9]: 296; Pang et al. [69]: 36; Nakano [16]: 157.

*Epilachna siphodentulata* Hoàng [54]: — an incorrect spelling used by Jadwiszczak & Węgrzynowicz [1]: 117.

*Ryszardia siphodenticulata* (Hoàng [54]): — Szawaryn et al. [34]: 563.

*Uniparodentata siphodenticulata* (Hoàng [54]): — Tomaszewska & Szawaryn [2]: 43.

**Distribution. Oriental.** China (Fujian, Guangxi, Guangdong, Guizhou, Hainan, Hunan) [69].
**Host plant. Rubiaceae:** *Randia oxyodonta* [16].

174) ***Uniparodentata subacuta* (Dieke, 1947)**

*Afissa subacuta* Dieke, 1947 [114]: 165. Type locality: China (Sichuan). — Liu [116]: 31.

*Epilachna subacuta* (Dieke [114]): — Pang & Mao [53]: 144; Kovář [94]: 628; Ren et al. [9]: 298; Jadwiszczak & Węgrzynowicz [1]: 119; Pang et al. [69]: 22, 36.

*Ryszardia subacuta* (Dieke [114]): — Szawaryn et al. [34]: 563.

*Uniparodentata subacuta* (Dieke [114]): — Tomaszewska & Szawaryn [2]: 43; Nakano [16]: 158.

**Distribution. Oriental**. China (Sichuan) [1,69].
**Host plant. Schisandraceae:** *Schisandra* cf. *propinqua* [16].

175) ***Uniparodentata szechuana* (Dieke, 1947)**

*Afissa szechuana* Dieke, 1947 [114]: 164. Type locality: China (Sichuan).

*Epilachna szechuana* (Dieke [114]): — Pang & Mao [53]: 144; Kovář [94]: 628; Ren et al. [9]: 298; Jadwiszczak & Węgrzynowicz [1]: 120; Pang et al. [69]: 36.

*Ryszardia szechuana* (Dieke [114]): — Szawaryn et al. [34]: 563.

*Uniparodentata szechuana* (Dieke [114]): — Tomaszewska & Szawaryn [2]: 43; Nakano [16]: 158.

**Distribution. Oriental**. China (Sichuan) [1,69].
**Host plant. Schisandraceae:** *Schisandra chinensis* [16].

176) ***Uniparodentata yongshanensis* (Cao & Xiao, 1984)**

*Epilachna yongshanensis* Cao & Xiao, 1984 [55]: 116, 128. Type locality: China (Yunnan). — Wang & Cao [91]: 120; Wang & Cao [122]: 118; Pang & Zeng [67]: 283; Jadwiszczak & Węgrzynowicz [1]: 131; Kovář [94]: 629; Ren et al. [9]: 300; Zhang & Ou [109]: 56; Pang et al. [69]: 36.

*Ryszardia yongshanensis* (Cao & Xiao [55]): — Szawaryn et al. [34]: 563.

*Uniparodentata yongshanensis* (Cao & Xiao [55]): — Tomaszewska & Szawaryn [2]: 43; Nakano [16]: 159.

**Distribution. Oriental**. China (Guizhou, Yunnan) [1,69].
**Host plants. Ranunculaeae:** *Clematis armandii*, *C. ranunculoides* [16,109].

## Data Availability

The data used in this study are based on published literature on the tribe Epilachnini, as well as the following online biodiversity platforms (GBIF, COL) and specialized databases (LadybirdBase and Coccinellidae South America), all of which are publicly available and cited in the reference list.
